# Plants Secondary Metabolites as Blood Glucose-Lowering Molecules

**DOI:** 10.3390/molecules26144333

**Published:** 2021-07-17

**Authors:** Mayadah Bashir Shehadeh, Ghadeer A. R. Y. Suaifan, Ala’ Mustafa Abu-Odeh

**Affiliations:** Department of Pharmaceutical Sciences, School of Pharmacy, The University of Jordan, Amman 11942, Jordan; gh.suaifan@ju.edu.jo (G.A.R.Y.S.); alaabuodeh82@gmail.com (A.M.A.-O.)

**Keywords:** diabetes, secondary metabolites, alkaloids, flavonoids, coumarins, insulin signal

## Abstract

Recently, significant advances in modern medicine and therapeutic agents have been achieved. However, the search for effective antidiabetic drugs is continuous and challenging. Over the past decades, there has been an increasing body of literature related to the effects of secondary metabolites from botanical sources on diabetes. Plants-derived metabolites including alkaloids, phenols, anthocyanins, flavonoids, stilbenoids, saponins, tannins, polysaccharides, coumarins, and terpenes can target cellular and molecular mechanisms involved in carbohydrate metabolism. In addition, they can grant protection to pancreatic beta cells from damage, repairing abnormal insulin signaling, minimizing oxidative stress and inflammation, activating AMP-activated protein kinase (AMPK), and inhibiting carbohydrate digestion and absorption. Studies have highlighted many bioactive naturally occurring plants’ secondary metabolites as candidates against diabetes. This review summarizes the current knowledge compiled from the latest studies published during the past decade on the mechanism-based action of plants-derived secondary metabolites that can target various metabolic pathways in humans against diabetes. It is worth mentioning that the compiled data in this review will provide a guide for researchers in the field, to develop candidates into environment-friendly effective, yet safe antidiabetics.

## 1. Introduction

Millions of years ago, plants appeared on land and prevailed in almost every habitat. Nearly 400,000 species have been recognized and assorted to date. In ancient medicine systems practiced worldwide, such as Ayurveda, Chinese and Egyptian, plants played a primary role in treating a myriad of diseases [1,2].

Nature contains an astronomical chemical space providing numerous compounds that impact human health and disease states. Over history, humankind has used natural products in crude form or as an extract. After the 20th revolution, scientists started to think about using pure isolated chemicals and elucidating their structures. The modern pharmaceutical industry has partially been founded by research looking for new therapeutic agents from medicinal plants [3].

Natural products are organic compounds synthesized by living organisms, e.g., plants, animals, and micro-organisms, which have biological activities. Secondary metabolites of botanical origin are produced to support and enhance the permanence of the plant. Photosynthesis, glycolysis and the Krebs cycle are overflowing with several intermediates that manufacture these metabolites [4,5].

Phytomedicine is a plant-based medicine, and it is one of the subsets of complementary and alternative medicinal (CAM) therapies [6]. The World Health Organization (WHO) defined herbal medicine as the knowledge, skills and practices based on the views and experiences related to different cultures used in the preservation of health and the prevention, diagnosis, improvement, or treatment of illness [2,7]. Traditional herbal medicines are plant-derived substances with minimal or no industrial processing that have been used to treat illness within local or regional healing conventionalisms [2].

Diabetes mellitus is a chronic progressive metabolic disorder of endocrine origin. It is characterized by disturbances of carbohydrate metabolism associated with hyperglycemia (WHO). Insulin is the key to maintaining an average level of blood glucose. In diabetes, insulin production is either absent or decreased, leading to hyperglycemia [6]. Diabetes is swiftly increasing: in 2019, approximately 463 million people had diabetes. According to the IDF (International Diabetes Federation) estimates, the number is expected to increase to 578 million in 2030 and 700 million in 2045 [8]. Diabetes mellitus can be treated by regulating the blood sugar level with different pharmacological and non-pharmacological approaches. Exercises, diet plans and diverse types of medications including insulin, glycosidase inhibitors, glycosuric, biguanides, meglitinides, sulphonylureas, thiazolidinediones and peptide analogs [6].

Published research has proven that several secondary metabolites demonstrate hypoglycemic activity in vivo and in vitro, and usually, they affect multiple targets, proteins and enzymes. Alkaloids, phenols, anthocyanin, flavonoids, saponins, tannins, terpenes and coumarins were found to elicit a significant influence on diabetes [9,10].

Phlorizin is a natural dihydrochalcone found in a number of fruitful trees, mainly the Malus genus. It produces renal glycosuria and blocks intestinal and renal glucose reabsorption by inhibiting the sodium-glucose symporters located there [11,12]. Recently, ertugliflozin arrived as the newest synthetic GLT-2 (glucose transporter-2) inhibitor to receive FDA approval for the treatment of diabetes [13,14].

This review highlights the proposed secondary metabolites mechanism-based action targeting various metabolic pathways involved in glucose metabolism in humans. The summarized data from in vivo and *in vitro* bioassays of phytochemicals will lead future research towards developing effective antidiabetics with low toxicity.

## 2. Mechanisms Involved in Glucose Metabolism and Homeostasis

Carbohydrates comprise the major constituents of a regular human diet. Starch digestion starts in the mouth and continues through the upper gastrointestinal tract until the intestine. It is converted into smaller molecules (monosaccharides) absorbed into the bloodstream [15].

Starch is mainly converted to glucose by the action of α- amylase that hydrolyzes macromolecules into oligoglucans, and α-glucosidase further degrades oligoglucans into absorbable glucose molecules at the brush border of the small intestine and is then absorbed through the glucose transporters sodium-dependent glucose cotransporter 1 (SGLT1) and glucose transporter 2 (GLUT2) [15,16]. Several phytoconstituents are known to suppress the activity of α-amylase, α-glucosidase and to inhibit intestinal absorption of glucose, inhibiting postprandial hyperglycemia and keeping the concentration of glucose in the blood constant after a meal [15,17].

**Figure 1:**
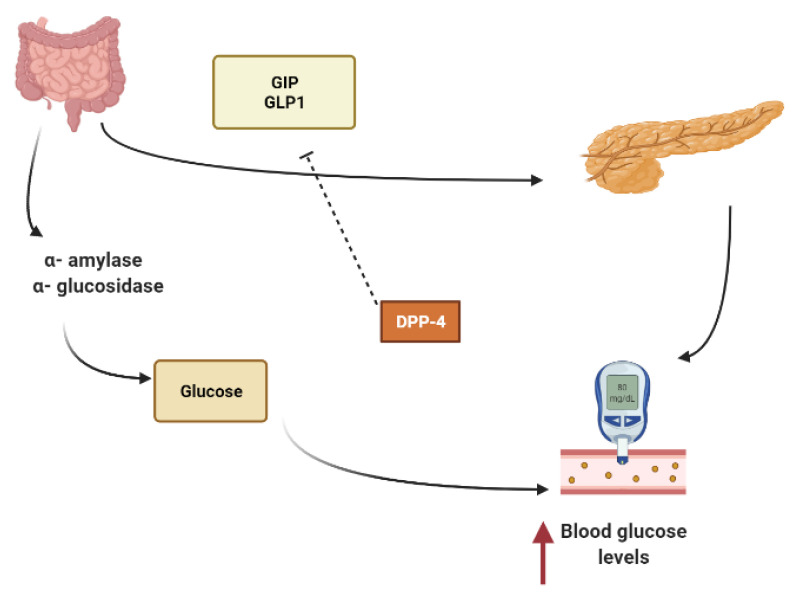
Carbohydrate metabolism in the gastrointestinal tract.

Other secondary metabolites can modulate the secretion of glucagon-like peptide-1 (GLP1), inhibiting dipeptidyl peptidase-4 (DPP4) from extending the effect of GLP1. In L-cells of the small intestine, basal serum GLP1 is released in response to nutrients loads (Figure 1). It promotes insulin secretion and stimulates the hypothalamus gland to induce postprandial satiety and to inhibit glucagon secretion. GLP1 has a very short half-life because it is hydrolyzed by dipeptidyl peptidase-4 (DPP4) [18,19,20].

**Figure 2:**
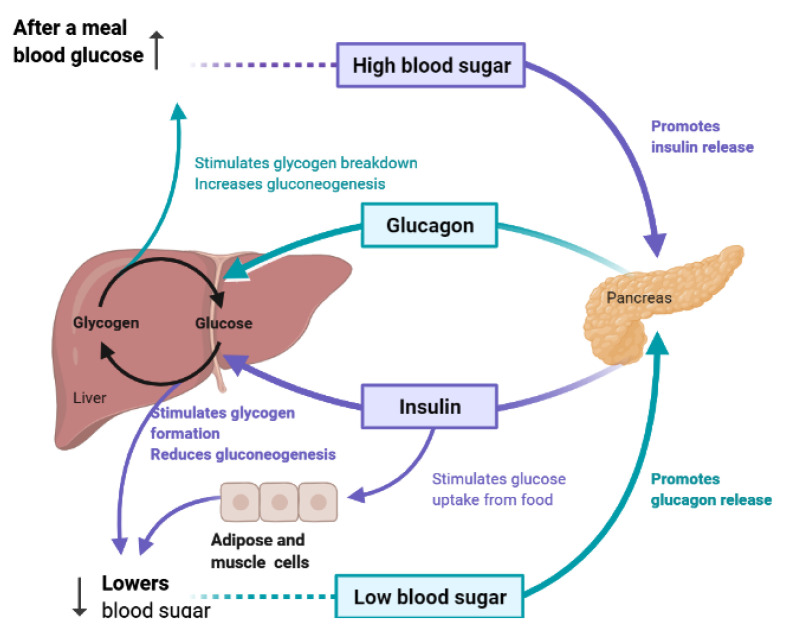
Carbohydrate balance.

To maintain constant blood glucose levels, various body organs, including the pancreas, liver, intestine, adipose and muscle tissues with various hormones and neuropeptides, work together. The pancreas plays a crucial role in glucose homeostasis by secreting insulin and its opponent glucagon [21]. The increased circulating glucose is sensed by pancreatic β-cells and subsequently glucose influx into the cells via GLUT2, an insulin-independent transporter (Figure 2). Glucose induces insulin secretion from β-cells via the closure of ATP-gated potassium channels and activation of voltage-gated calcium channels [16,21,22,23].

**Figure 3:**
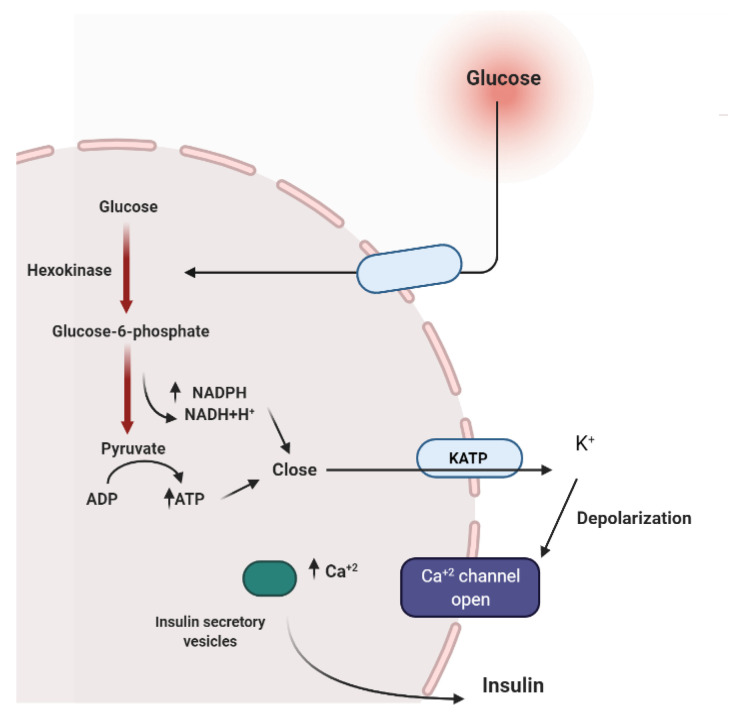
Mechanism of insulin exocytosis.

Several medicinal plants constituents can affect insulin secretion via closing the ATP-sensitive potassium channel (KATP), acting on the Ca^2+^ channels. Others can decrease insulin degradation by inhibiting insulinase or by possessing cAMP phosphodiesterase inhibitory activity (Figure 3) [22].

## 3. Insulin Signal Transduction

Insulin modulates several metabolic pathways through a cascade of signal transduction steps initiated when the insulin binds to the insulin receptor (IR), stimulating a receptor’s intrinsic kinase activity [21]. Insulin receptor substrates IRS1 and IRS2 are phosphorylated by several kinases such as ERK (extracellular signal-regulated kinases), JNK (c-Jun N-terminal kinase), and AMPK (AMP-activated protein kinase). Consequently, insulin substrates activate a number of kinases, including PI3K (PI3K: phosphoinositide 3-kinase)/Akt pathway as well as the MAPK (MAPK: mitogen-activated protein kinase) pathway [24,25].

**Figure 4:**
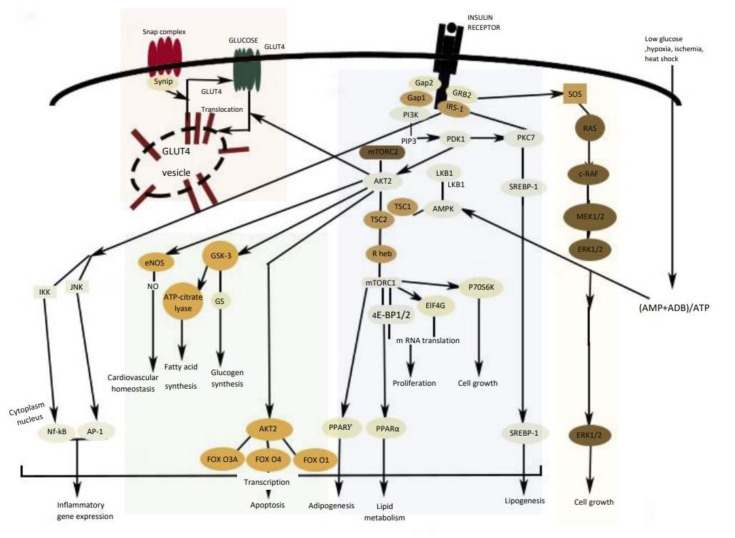
Insulin signal transduction, 4E-BP1/2: eukaryotic translation initiation factor 4E binding protein/threonine-protein kinase; AMPK: AMP-activated protein kinase α; AP-1: activator protein 1 transcription factor; c-Raf: RAF proto-oncogene serine/threonine-protein kinase; DEPTOR: DEP domain-containing mTOR-interacting protein; eIF4G: eukaryotic translation initiation factor 4 gamma; eNOS: endothelial nitric oxide synthase; Erk1/2: extracellular signal-regulated kinases 1/2; FoxO: forkhead box O transcription factors; Gab: GRB2-associated-binding protein; GLUT2 or 4: glucose transporter type 2 or 4; GPR120: G-protein coupled receptor 120; GRB2: growth factor receptor-bound protein 2; GS: glycogen synthase; GSK-3: glycogen synthase kinase 3; GβL: G protein beta subunit-like; IKK: IκB kinase; IRS-1: insulin receptor substrate 1; JNK: c-Jun N-terminal kinase; LKB1: liver kinase B1; MEK1/2: mitogen-activated protein kinase 1/2; mTORC: mechanistic target of rapamycin; NF-κB; nuclear factor kappa-light-chain-enhancer of activated B cells; NO: nitrogen oxide; p70S6K: p70S6 kinase PDK1 phosphoinositide-dependent kinase-1; PI3K: phosphoinositide 3-kinase; PKCζ: protein kinase C zeta type; PPAR: peroxisome proliferator-activated receptor; PRR5: proline-rich protein 5; Raptor: regulatory-associated protein of mTOR; Rheb: Ras homolog enriched in brain; Rictor: rapamycin-insensitive companion of mammalian target of rapamycin; RIP: receptor-interacting protein kinases; Sin1: stress-activated map kinase interacting protein 1; SNARE: soluble NSF attachment protein receptor; SOS: son of sevenless protein; SREBP-1: sterol regulatory element-binding protein 1; Synip: syntaxin 4-interacting protein; TAB-1: TGF-beta activated kinase 1.

The IRS protein activates phosphatidylinositol 3 kinase (PI3K) that causes the activation of Akt, the main mediator to activate the most biochemical mechanism in glucose metabolism [26]. Akt activates the glucose transporter translocation to the cellular membrane (GLUT-4) and triggers the phosphorylation of glycogen synthase kinase 3 (GSK3), which leads to stimulation of glycogen synthesis in liver and skeletal muscle and downregulation of PEPCK (phosphoenolpyruvate carboxykinase) and G6Pase (glucose-6- phosphatase) gene expression [24]. However, in a diabetic state, these pathways are not blocked because insulin is unable to adequately regulate the gene expression and function of PEPCK and G6Pase, leading to excessive hepatic glucose production through gluconeogenesis and glycogenolysis [9,16]. In addition, the activation of the Akt system leads to protein synthesis through the activation of the mechanistic target of rapamycin complex 1 (mTORC1), cell survival by the suppression of various pro-apoptotic molecules, mainly of the FOXO (forkhead box O) family of transcription factors (Figure 4) [27].

In the liver, insulin activates AMPK which leads to the inhibition of acetyl-CoA carboxylase (ACC) and sterol regulatory element-binding protein (SREPB-1) activity, thereby inhibiting fatty acid biosynthesis and increasing fatty acid oxidation [24].

MAPK is a specific protein kinase involved in various physiological and biochemical mechanisms, including cell differentiation, proliferation, apoptosis, and cell endurance. ERK1/2 (extracellular-signal-regulated kinase 1/2) and JNK (c-Jun N-terminal kinase) are other cell signaling kinases co-task with MAPK, involved in cell growth, differentiation, inflammatory response, and apoptosis. Overstimulation of MAPK generally provides the failure of insulin synthesis linked with the apoptosis process in pancreatic islet cells and downregulates GLUT4 expression [25,28].

## 4. Liver and Glucose Homeostasis

**Figure 5:**
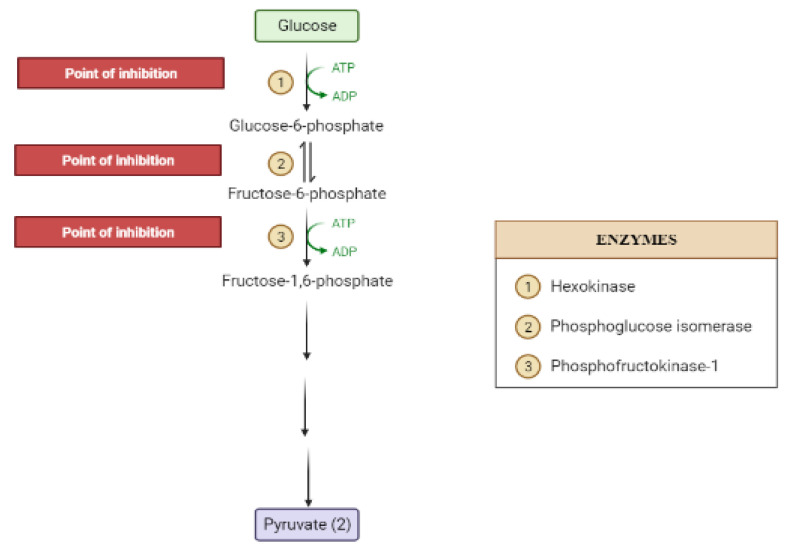
Glycolysis.

**Figure 6:**
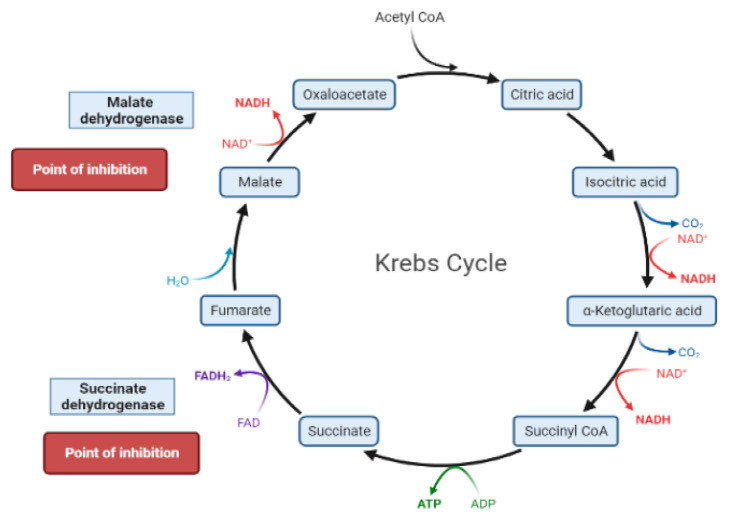
Krebs cycle.

The liver is the chief organ in maintaining glucose homeostasis through controlling various pathways, including glycogenesis, glycogenolysis, glycolysis and gluconeogenesis [29]. During feeding conditions, glucose is a primary fuel source across multiple fuel sources across numerous body tissues by eliciting ATP molecules during hydrolysis and the Krebs cycle. The increase in glucose uptake in hepatocytes promotes glycolysis and lipogenesis to generate triglycerides as storage forms of fuel [22,29]. Glycolysis can be regulated through the reactions catalyzed by hexokinase, phosphofructokinase, and pyruvate kinase (Figure 5 and Figure 6). Moreover, there are seven enzymes involved in this Krebs cycle, of which only two enzymes, succinate dehydrogenase and malate synthase, can be regulated. Several plant secondary metabolites (discussed below) can regulate and affect these enzymes except for the pyruvate kinase enzyme [22,30,31].

**Figure 7:**
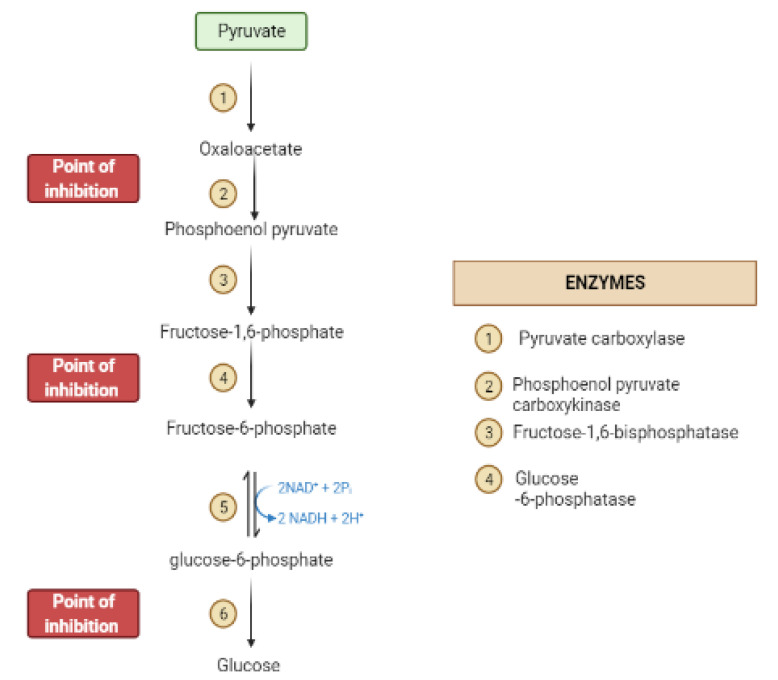
Gluconeogenesis.

During the fasting state, the liver is the leading factory for glucose production through gluconeogenesis and glycogenolysis. The main regulatory enzymes in the gluconeogenesis pathway include glucose 6-phosphatase (G6Pase), fructose 1, 6-bisphosphatase (Fbpase1), PC (pyruvate carboxylase), and phosphoenolpyruvate carboxykinase (PEPCK), all can be inhibited by plant secondary metabolites (Figure 7) [29,30].

**Figure 8:**
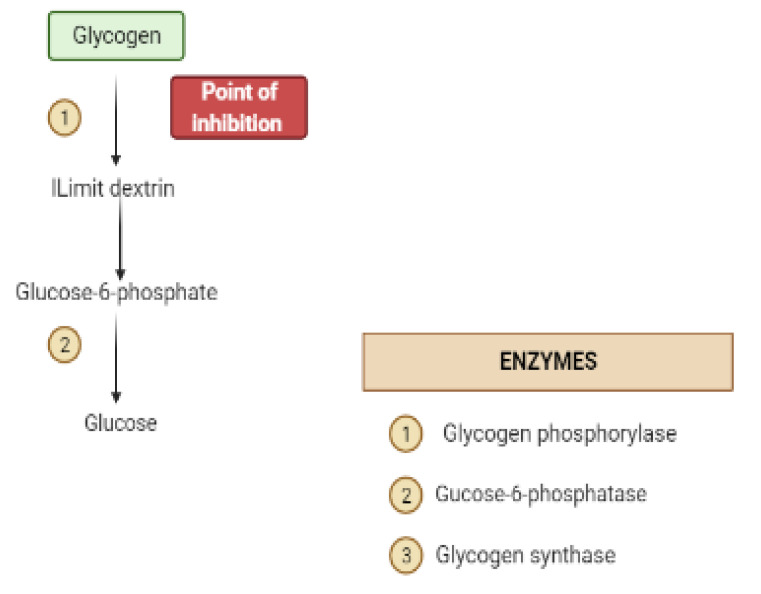

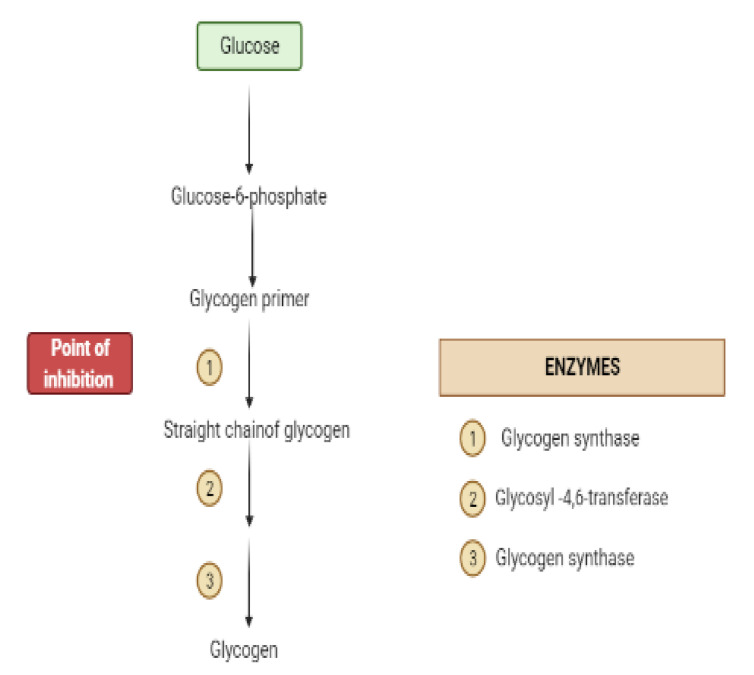
Glycogen metabolism, synthesis and hydrolysis.

Glycogen synthesis from unused glucose is a multistep process carried out by the enzyme glycogen synthase in the liver. Secondary metabolites can affect the process through glycogen synthase. In addition, glycogenolysis is inhibited through glycogen phosphorylase (Figure 8) [22].

## 5. Obstacles for Insulin Signal Transduction and Insulin Effects

Several obstacles can affect the signal transduction of insulin in different tissues. Protein tyrosine phosphatase 1B (PTP1B) is a known negative regulator of the insulin-stimulated signal transduction pathway. PTP1B is localized on the cytoplasmic surface of the endoplasmic reticulum in classical insulin targeted tissues such as the liver, muscle, and fat. PTP1B catalyzes the dephosphorylation of activated insulin receptors (IR), resulting in down-regulation of insulin signaling [31,32].

The increase in the free fatty acids is connected to alteration in the diacylglycerol (DAG)/protein kinase C (PKC) pathway. The rise in DAG activates PKC-θ, -β2 and –δ that phosphorylates IRS1, which interferes with insulin-stimulated phosphorylation of IRS1, thus inhibiting insulin signaling. It is believed that targeting PKC is beneficial in treating type 2 diabetes as it will address the secretory defect promoting insulin secretion [33,34].

Inflammatory mediators (such as TNF-α, Il-6) can impair insulin and promote serine phosphorylation of IRS-1 impairing insulin signaling or can cause degradation of IRS. It can reduce GLUT-4 expression, decreasing glucose entry to cells and cause inflammation-induced nitric oxide release that suppresses PI3K–Akt pathway [23]. It is well-known that inflammation is a crucial cause for diabetes type-2. Relieving inflammation by secondary metabolites can improve diabetes [35].

Oxidative stress has been linked to diabetes. It comprises insulin secretion and insulin action. It activates NF-κB and JNK, IRS degradation, suppresses GLUT-4 expression and translocation, activates inflammatory responses [36,37]. Plant secondary metabolites can stimulate mitochondrial metabolism and/or decrease mitochondrial dysfunction through targeting sirtuin 1 activators (SIRT1) and PPAR α [37].

A dependent relationship exists between insulin and mitochondria. Insulin release depends on mitochondrial ATP production, and mitochondrial fusion depends on insulin. Mitochondria is a major source of free radicals. However, overproduction of free radicals causes mitochondrial dysfunction [37,38].

## 6. Skeletal Muscle and Adipose Tissue

**Figure 9:**
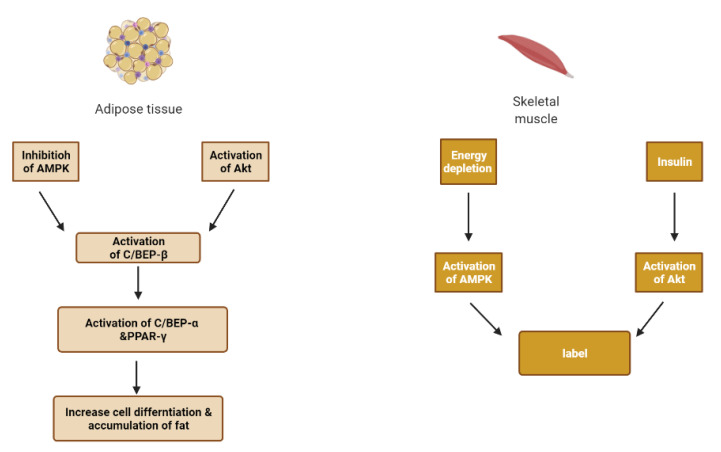
Insulin effect on adipose tissue and muscle tissue.

Insulin works on adipose tissue and skeletal muscle tissue where it causes glucose uptake, use and storage by skeletal muscle (Figure 9). Glucose uptake is mediated by the combined influence of glucose concentrations, insulin signaling, insulin receptor substrate (IRS)-1/phosphatidylinositol kinase (PI3K)/kinase B (or Akt) pathway, and increasing membrane localization of transporters such as glucose transporter 4, GLUT4 [16,24].

Adipose tissue plays a major role in the ability of the body to sense insulin. When nutrient intake exceeds the capacity of fat cells to store excess calories, hypoxia appears in adipose tissue. Subsequently, hypoxia-inducible factor-1 (HIF-1) is activated, and the expression of c-Jun N-terminal kinase (JNK) and inhibitor nuclear factor kappa-B kinase (IKK) is increased, producing inflammation in adipose tissue [33].

With the aggravation of inflammation, many inflammatory cytokines are released to exacerbate insulin resistance and lipolysis, reduce the activity of peroxisome proliferator-activated receptor γ (PPARγ), and accelerate the fat cell death and inflammation [33].

A vicious cycle is formed due to decreased insulin activity as an anti-lipolytic hormone and hyperinsulinemia that activates the lipoprotein lipase and causes free fatty acid (FFA) release from lipoproteins [33].

A high level of FFA causes the accumulation of diacylglycerol (DAG) and ceramide. DAG can inhibit insulin action by activating the protein kinase C isoforms (PKC) and interfering with insulin signal transduction by serine phosphorylation of insulin receptors (IRS) [33,39]. Ceramide is a potent activating agent of inflammation. It can activate JNK and NF-κB/IKK, which are closely related to insulin resistance. It can induce pancreatic β-cell apoptosis and reducing insulin gene expression [33,40].

Additionally, fat deposits in the liver and is accompanied by the build-up of insulin resistance. Kupffer cells in the liver are activated to release inflammatory cytokines. Consequently, nuclear factor κB (NF-κB) is activated and regulates the inflammatory state [33].

Lipid peroxidation and glucose autoxidation give rise to the accumulation of reactive oxygen species (ROS) and free radicals. Thereby, increased active oxygen groups result in hepatic oxidative injury and jams of antioxidant enzymes such as SOD (superoxide dismutase), CAT (catalase) and GP (glutathione peroxidase), which further aggravate the progression of insulin resistance and pancreatic β-cell dysfunction [33].

Peroxisome proliferator-activated receptor gamma (PPAR) is expressed in several tissues, especially macrophages, adipose, muscle and mainly involved in adipocyte differentiation. The PPARs and retinoid X receptors regulate some genes involved in lipid and carbohydrate metabolism. There are three human isoforms of PPAR; α, δ and γ [15].

PPARγ plays a key role in adipogenesis and glucose regulation. Activated PPAR γ binds to retinoic acid X receptor to regulate transcriptional activation of downstream target genes linked to diabetes. In addition, it is also reported to enhance GLUT-1 and GLUT-4 translocation in the liver and skeletal muscle. It also potentiates insulin sensitivity by reducing TNF-α and elevating adiponectin expression. On the other hand, PPARα is a regulator of fatty acid catabolism and peroxisome proliferation in the liver [15]. Table 1 summarizes the biological effects of insulin in normal and diabetic status [41].

## 7. Methodology

The key words of: diabetes, plant secondary metabolites, alkaloids, phenols, anthocyanins, flavonoids, stilbenoids, saponins, tannins, polysaccharides, coumarins and terpenes, were searched for in the Pub-Med, SciFinder, EBSCO, ScienceDirect and Google-scholar databases. Relevant papers in the English language from the year 2010 up to 2021 were collected. To qualify for inclusion in this review, publications should have reported biological and chemical data for isolated secondary metabolites with in vitro or in vivo test models of type 2 diabetes.

## 8. Result and Discussion

### 8.1. Secondary Metabolites and Antidiabetic Activity

#### 8.1.1. Alkaloids

**Figure 10:**
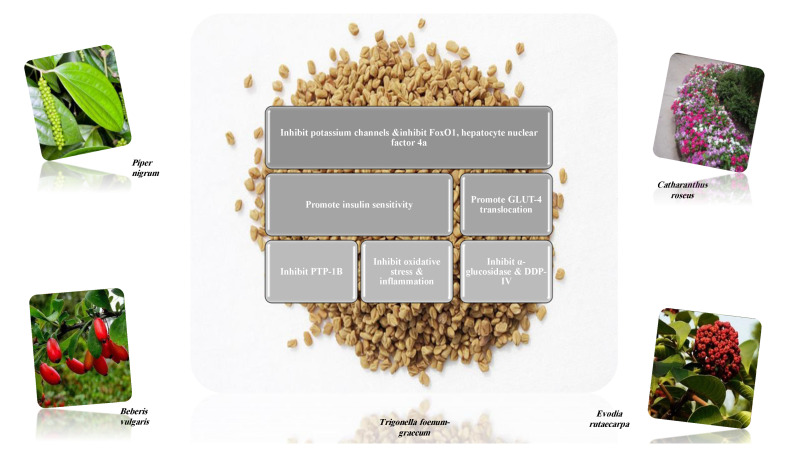
Antidiabetic activity of naturally occurring alkaloids.

Alkaloids are a remarkably diverse group of nitrogen-containing compounds with limited allocation in the plant kingdom, especially in angiosperms. They may contain one or more nitrogen atoms within a heterocyclic ring and are classified as pyrrolidine, pyridine, quinoline, isoquinoline, indole, quinazoline, steroidal, diterpenoid, and other alkaloids. They have a wide spectrum of biological activities, including hypoglycemic. The therapeutic impact of alkaloids against diabetes is mediated through various pathways and signaling cascades (Figure 10) [27,42,43,44].

Alkaloids are considered eminent inhibitors of α-glucosidase and α- amylase enzymes, one of the most effective approaches to decrease the level of blood glucose in type 2 diabetes, for example, broussonetine and radicamine [45,46]. Alkaloids inhibit dipeptidyl peptidase-4 (DDP-4), for example, bebeerine that degrades gut incretin hormones, including glucagon-like peptide-1 (GLP-1) and gastric inhibitory polypeptide (GIP). A peptide that induces β-cell differentiation stimulates insulin biosynthesis and release, inhibits gastric emptying, directly reducing food intake and prevents glucagon release from the α-cells of the islets of Langerhans [20,47,48].

Adenosine monophosphate (AMP) activated protein kinase is a cellular fuel sensor and glucose transporter regulator. It stimulates glucose uptake and modulates insulin secretion. Several alkaloids are allosteric activators of AMPK and enhance its enzymatic activity [45,49].

Alkaloids increase glucose-4 transporter translocation, increase insulin secretion via regenerating the pancreatic β-cells and enhanced glucose-stimulated insulin secretion (GSIS) rather than basal insulin secretion [45]. Examples of hypoglycemic alkaloids and their proposed mechanisms of action (Table 2). Several in vitro and in vivo studies were designed to explore the antidiabetic activity of different isolated alkaloids (Table 3).

On the other hand, some were reported to trigger insulin secretion such as bisbenzylisoquinoline, quinolizidine, isoquinoline, indole and pyridine alkaloids [46].

Several plant families: Acanthaceae, Apocynaceae, Bignoniaceae, Campanulaceae, Euphorbiaceae, Fabaceae, Moraceae, Piperaceae, Portulacaceae, Ranunculaceae, and Rutaceae, were investigated for their alkaloids-rich parts. All exhibited various mechanisms of action for their antidiabetic activity. The most frequent mechanism was the digestive enzymes inhibitory activity [42].

Alkaloids have diverse and variable structures. They inhibit enzymes at a lower dose compared to other metabolites. They affect multiple targets to lower hyperglycemia, but α-glucosidase inhibition seems to be the most promising mechanism [46,47,48,50].

Regrettably, the toxicity of some alkaloids, instability under physiologic pH, hence low bioavailability and short half-life, may limit their clinical use. However, new pharmaceutical delivery techniques can overcome the issues. Further investigation is needed to explore their pharmacological mode of action in-vivo and in clinical setting [46,50].

**Table 2:** Proposed antidiabetic effects of different alkaloids.

Name of Compound	Chemical Structure	Type of Alkaloid	Mechanism	References
Berberine	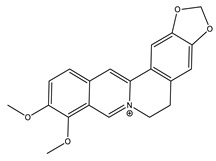	Isoquinoline	✓ Increases glucose uptake via activation of AMPK ✓ Inhibits protein tyrosine phosphatase 1B (PTP1B) ✓ Inhibits DDP-4 enzyme ✓ Increases the secretion of glucagon-like peptide 1 (GLP-1) ✓ Improves insulin sensitivity through adipokines and leptin ✓ Stimulates glucose uptake by the up-regulation of GLUT-1 expression ✓ Suppresses oxidative stress	[27,48,51,52]
Evodiamine	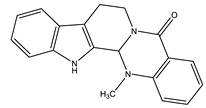	Quinolone	✓ Improves insulin resistance through affecting AMPK, PPAR-γ	[27,46]
Glycosin	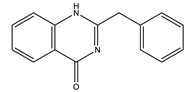	Quinazoline	✓ Interacts with DPP-IV, insulin receptor and PTP-1B and PPARγ	[46]
Lupanine	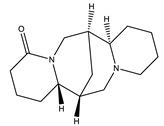	Quinolizidine	✓ Inhibits KATP channels and increasing the expression of the Ins-1 gene ✓ Enhances glucose-induced insulin release	[45,46]
Neferine	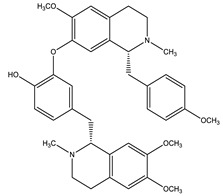	Isoquinoline	✓ Enhances insulin sensitivity	[27]
Piperine	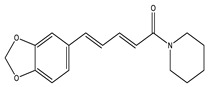	Piperidine	✓ Reverses insulin resistance ✓ Restores plasma insulin, concentration through affecting AMPK and adiponectin ✓ Lowers inflammatory cytokines IL-1β, TNF-α levels and NF-κB	[27]
Oxymatrine	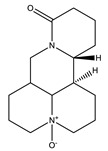	Quinolizidine	✓ Protects tissue architecture of the pancreas and liver	[27,47]
Trigonelline	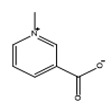	Pyridine	✓ Increases the glucokinase/glucose-6-phosphatase (G6Pase) ratio ✓ Increases GLUT4 expression and translocation ✓ Increases serum insulin level ✓ Suppresses β-cell damage and augmentation of β-cell regeneration ✓ Suppresses oxidative stress	[27,48]
Vidoline	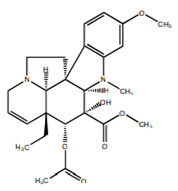	Indole	✓ Increases insulin secretion ✓ Inhibits protein tyrosine phosphatase-1B (PTP1B) activity	[27,48]

**Table 3:** In vitro, in vivo and in silico models for different isolated alkaloids.

Isolated Compound	Type of Alkaloids	Mechanism	Dose of the Tested Compound (Duration)	Model	References
Berberine	Isoquinoline	Increased mRNA and protein expressions of GLUT-4 and reduced activation of the hypothalamus-pituitary-adrenal axis.	200 mg/kg (4 weeks)	High-fat diet and streptozocin-induced diabetic rats	[50]
		Modulated gut microbiota.	200 mg/kg (8 weeks)	High-fat diet obese rats	[51]
		Inhibited expression of the gluconeogenic proteins (PEPCK and G-6-Pase) in the liver.	156 mg/kg (12 weeks)	High-fat diet and streptozocin-induced diabetic rats	[52]
		Increased expression of skeletal muscle GLUT- 4, mRNA had antioxidant activity.	50, 100 mg/kg (6 weeks)	High- fat and glucose diet hamsters	[53]
Conophylline	Vinca	Pancreatic β-cells regenerator.	0.1 µg/mL	In vitro: ICC cell line	[54]
Coptisine	Isoquinoline	PTP1B inhibition.	6.25–50 µM	In vitro: enzymatic assay and in silico	[55]
Ephedrine	Phenylalanine derived	DDP-4 inhibition, IC_50_ was 124 µM	10^−5^–10^−3^ M	In vitro: binding assay and in silico	[56]
Evodiamine	Quinolone	Activated AMPK phosphorylation.	0.1, 1 mg/kg (6 months)	Ageing mice model	[57]
Koenidine	Carbazole	GLUT-4 translocation.	25, 50 µM	In vitro: L6-GLUT4myc myotubes cell line	[58]
Lupanine	Quinolizidine	Potentiated insulin release by directly affecting KATP channels.	0.5 mmol/L 20 mg/kg	In vitro: NS-1E cell line Streptozocin-induced diabetic mice	[59]
Magnoflorine	Aporphine	PTP1B inhibition.	12.5 to 100 μM	In vitro: enzymatic assay and in silico	[55]
Neferine	Isoquinoline	Upregulated GLUT-4 expression and plasma membrane fusion.	150 µM	In vitro: L6 cell line	[60]
Nigelladine	Norditerpene	Reduce PTP1B overexpression, promote glycogen synthesis and activated the PI3 K/Akt signaling pathway.	50 µM	In vitro: L6 cell line	[61]
Nuciferine	Aporphine	Insulin secretion stimulator.	10, 20 mM (24 h)	In vitro: INS1-E cell line	[62]
Picrasidine	Cathinone	PTP1B inhibition	IC_50_: 19.80 ± 0.62 µM	In vitro: Hepatocellular carcinoma (HepG2) cell line	[63]
Piperine	Piperidine	Down-regulation of mRNA levels of pro-inflammatory cytokines.	40 mg/kg (10 weeks)	Monosodium glutamate diabetic mice	[64]
		Ameliorated dysfunction of β-cell.	15, 30 mm/kg (8 weeks)	High-fat-induced diet mice	[65]
Sanguinarine	Benzo-phenanthridine	Activation of AMPK.	0.2, 1, 10 µM (1 h)	In vitro: cell based assay and in silico	[66]
Trigonelline	Pyridine	Reduction of insulin resistance through PAR-γ/GLUT4-leptin/TNF-α signaling pathway.	40 mg/kg (8 weeks)	High-fat diet and Streptozocin-induced diabetic rats	[67]
		Protection of β-cells Exhibited antioxidant activity.	40 mg/kg (8 weeks)	High-fat and high fructose-induced diabetic rat	[68]
Vindolicine	Indole	PTP1B inhibition and induction of glucose uptake, IC_50_ was 73.5 ± 11.3	12.5, 50 µg/mL (24 h)	In vitro: β-TC6 and C2C12 cell lines	[69]
Vindolinine	Indole	PTP1B inhibition and induction of glucose uptake	12.5, 50 µg/mL IC_50_: 57.6 ± 10.7 µM (24 h)	In vitro: β-TC6 and C2C12 cell lines	[69]
Vindolidine	Indole	PTP1B inhibition and induction of glucose uptake	12.5, 50 µg/mL IC_50_: 180.1 ± 19.0 µM (24 h)	In vitro: β-TC6 and C2C12 cell lines	[69]
Vindogentianine	Indole alkaloid	PTP1B inhibition and induction of glucose uptake	12.5, 25, 50, 100 and 200 μg/mL IC_50_: more than 50 μg/mL (24 h)	In vitro: β-TC6 and C2C12 cell lines	[70]

#### 8.1.2. Dietary Phenols

Phenolics are a complex category of bioactive secondary metabolites produced through shikimate and acetate pathways. Phenols are classified into four major groups: simple phenols or phenolic acid, coumarins, lignans, lignins, stilbenes, hydrolyzable and condensed tannins and flavonoids which include flavans, flavonols, flavones, flavanones, isoflavones, and anthocyanins [10,71]. Simple phenols have only one aromatic carboxylic acid with hydroxyl derivatives and two types; hydroxybenzoic acid and hydroxycinnamic acid derivatives. Several simple phenolic acids were assessed clinically and in vivo for their antidiabetic activities. The considerable hypoglycemic activity was shown for several hydroxycinnamic acid derivatives listed in (Table 4) [10].

**Figure 11:**
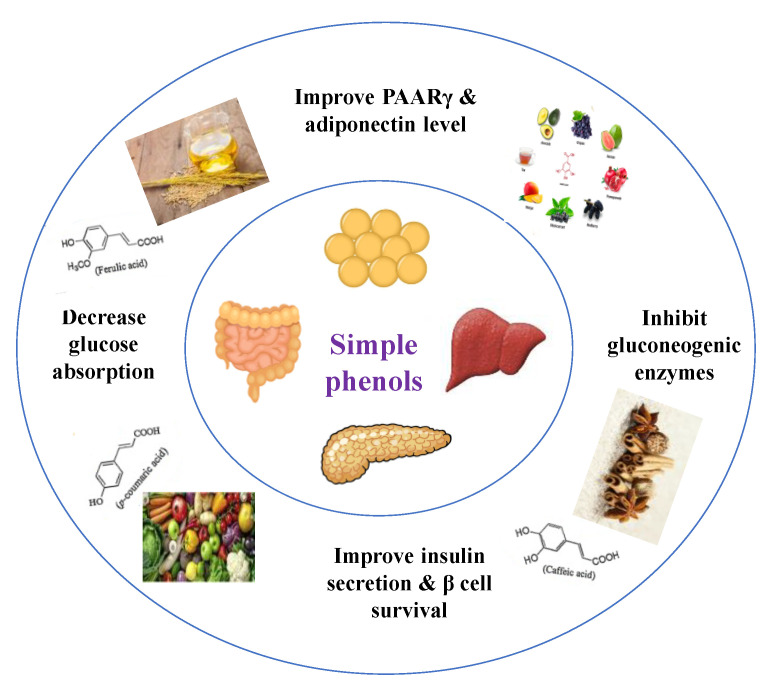
Antidiabetic activity of simple phenols.

Dietary phenols have positive health effects in diabetes via protection of pancreatic islet β-cell, reduction of β-cell apoptosis, promotion of β-cell proliferation, attenuation of oxidative stress, activation of insulin signaling, stimulation of pancreas to secrete insulin, inhibition of glucose absorption, regulation of intestinal microbiota, modification of inflammation response, and inhibition of the formation of advanced glycation end-products (Figure 11) [10].

The efficacy of phenols depends primarily on their bioavailability and metabolism after entering the body. Phenolics exhibited a magnificent power to defeat diseases due to their antioxidant potential. They have direct and indirect antioxidant activity by inducing endogenous protective enzymes and positive regulatory effects on signaling pathways. Their antioxidant power depends on the presence of esterified or glycosylated substitution [71,72]. Among all mechanisms reported for phenolic acids to manage diabetes, the best was the inhibition of postprandial hyperglycemia and the antioxidant activity, as diabetes is known as an oxidative stress disorder [71]. Table 5 shows different studied mechanisms of simple phenolics.

In a randomized cross-over trial in healthy overweight men, decaffeinated coffee consumption and chlorogenic acid intake were associated with reducing early glucose and insulin responses during two hours in oral glucose tolerance test (OGTT) [73,74].

**Table 4:** Proposed antidiabetic effects of different simple phenols.

Name of Compound	Chemical Structures	Mechanism	References
Caffeic acid	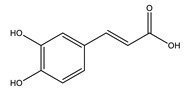	✓ Improves insulin level ✓ Improves β-cell survival ✓ Suppresses hepatic glucose production	[10,75]
p-Coumaric acid	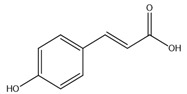	✓ Activates AMPK activity ✓ Enhances antioxidant potential and anti-inflammation effects ✓ Increases insulin sensitivity ✓ Inhibits adipogenesis and gluconeogenesis ✓ Improves β-cell function ✓ Modulates glucose metabolism enzymes ✓ Reduces the intestinal absorption of carbohydrate ✓ Stimulates insulin secretion	[10,76]
Cinnamic acid	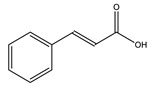	✓ Decreases the activity of DDP-4 ✓ Decreases PTP-1B ✓ Decreases α-amylase and glucosidase ✓ Decreases gluconeogenesis ✓ Increases glycolysis ✓ Increases the expression of PPARγ ✓ Increases adiponectin level ✓ Stimulates insulin secretion	[75,77]
Chlorogenic acid	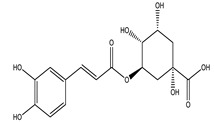	✓ Attenuates intestinal glucose absorption ✓ Activates AMPK pathway ✓ Improves insulin sensitivity ✓ Modulates oxidative stress	[75,78]
Ellagic acid	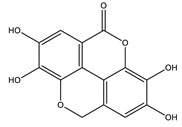	✓ Increases gene expression of insulin in β-cell ✓ Suppresses oxidative stress and inflammation	[75,79]
Ferulic acid	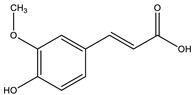	✓ Decreases glucose-6-phosphatase and phosphoenolpyruvate carboxykinase activities in liver ✓ Increases glucokinase activity	[10,77]
Gallic acid	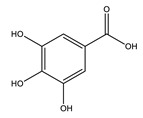	✓ Alleviates oxidative stress ✓ Decreases gluconeogenesis ✓ Increases insulin secretion ✓ Increases glycolysis ✓ Improves β cell regeneration ✓ Promotes glucose transporter GLUT-4 translocation ✓ Suppresses nuclear factor (NF)-κB activity and cytokine release ✓ Up regulation of PPARγ expression and Akt activation	[75,76]
Hydroxycinnamic acid	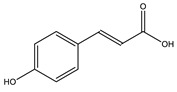	✓ Activates AMPK activity ✓ Antioxidant potential and anti-inflammation effects ✓ Inhibits adipogenesis and gluconeogenesis ✓ Inhibits glucose-6-phosphatase, phosphoenolpyruvate carboxykinase ✓ Promotes glucokinase activity ✓ Promotes 𝛽-cell function	[10,77]
Protocatechuic acid	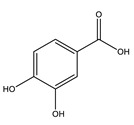	✓ Inhibits α-amylase and glucosidase activity ✓ Enhances GLUT4 translocation ✓ Enhances adiponectin secretion ✓ Enhances the expression of PPARα and PPARγ ✓ Increases the activity of hexokinase, glucose-6-phosphate dehydrogenase ✓ Reduces the activity of glucose 6-phosphatase (G6Pase), fructose-1,6-bisphosphatase, and sorbitol dehydrogenase	[75,80]
Syringic acid	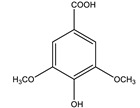	✓ Promotes secrete insulin from existing islet β-cells ✓ Augmentation of glucose utilization by peripheral tissues ✓ Stimulates the β-cell regeneration	[75,81]

**Table 5:** In vitro, in vivo and in silico models of different isolated simple phenols.

Compound	Mechanism	Dose of the Tested Compound (Duration)	Model	References
Caffeic acid	Exhibited antioxidant effect.	40 mg/kg (4 weeks)	Nicotinamide-streptozocin-induced diabetic mice	[82]
	Exhibited antioxidant effect, increased insulin secretion and protected pancreatic cells.	25, 35 mg/kg (5 weeks)	Streptozocin-induced diabetic rats	[83]
Coumaric acid	Activation of pancreatic GLUT-2, increased level of insulin, decreased gluconeogenic enzymes (glucose-6-phosphatase and fructose-1, 6-bisphosphatase).	100 mg/kg (4 weeks)	Streptozocin-induced diabetic rats	[84]
	Decreased the level of TNF-α, increased the levels of PPARγ mRNA and adiponectin.	40 mg/kg (6 weeks)	Streptozocin-induced diabetic rats	[85]
	Exhibited antioxidant effect, increased insulin level, protected pancreatic cells.	100 mg/kg (4 weeks)	Streptozocin-induced diabetic rats	[86]
Cinnamic acid	Stimulated glucose-induced insulin secretion	5, 10 mg/kg	Streptozocin-induced diabetic rats	[87]
Chlorogenic acid	Increased expression of adiponectin receptors, increased phosphorylation of AMPK in the liver and muscle, increased mRNA and protein levels of PPAR-α in the liver.	80 mg/kg (12 weeks)	Diabetic mice	[88]
Ellagic acid	Activated insulin signaling pathway in the liver by increasing phosphorylated Akt had an antioxidant effect	50 mg/kg (28 days)	Insulin resistant diabetic rats	[89]
Ferulic acid	Improved hepatic glycogenesis by phosphorylating and inhibiting GSK3β, suppressed gluconeogenesis by phosphorylating FoxO1, Reduced IRS1, PKC-ε and PTP1B, which are known to inhibit the insulin signaling.	50 mg/kg (4 weeks)	High-fat and fructose-induced diet diabetic rat	[90]
	Reduced GLUT-2 expression.	50 mg/kg (4 weeks)	High-fat and fructose-induced diet diabetic rat	[91]
Gallic acid	Decreased the level of TNF-α, increased the levels of PPARγ mRNA and adiponectin.	20 mg/kg (6 weeks)	Streptozocin-induced diabetic rats	[85]
Protocatechuic acid	Exhibited antioxidant effect, improved hepatic insulin resistance by modulating IRS1/PI3K/AKT2 pathways.	100 mg/kg	Streptozocin-induced diabetic rats	[92]
	Stimulated insulin signaling pathway increasing GLUT4 translocation and glucose uptake.	1–150 µmol/L	In vitro: human visceral adipocytes	[93]
	Attenuated the increase in the expression of gluconeogenic enzymes, restored AKT protein phosphorylation (restores GLUT-4 translocation).	50, 100 mg/kg (14 days)	Dexamethasone diabetic rats	[94]
Syringic acid	Ameliorated the functional and histological abnormalities and hepatic mitochondria biogenesis (fight insulin resistance).	25, 50 and 100 mg/kg (6 weeks)	Streptozocin-induced diabetic rats	[95]

#### 8.1.3. Anthocyanins

**Figure 12:**
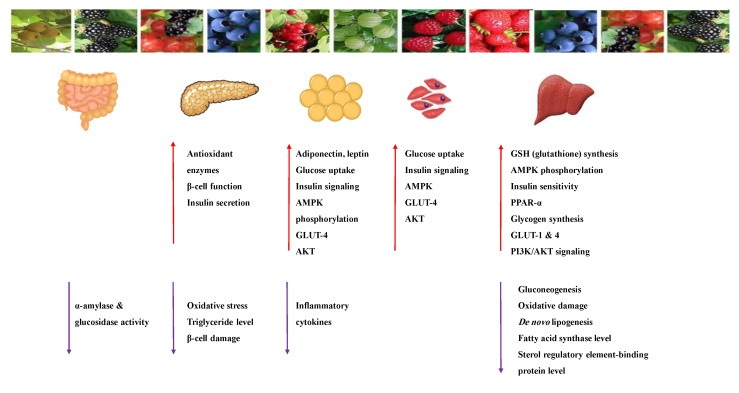
Anthocyanins and their protective role against diabetes.

Anthocyanins are widespread natural polyphenolic pigments in the plant kingdom. Six anthocyanidins are commonly synthesized in plants: cyanidin, delphinidin, malvidin, peonidin, pelargonidin and petunidin [96]. Table 6 shows the proposed mechanisms of anthocyanins as antidiabetic agents. They have valuable health effects documented in many in vivo and in vitro studies (Table 7). It appears that they affect several signaling pathways of glucose metabolism (Figure 12) [21,97,98,99].

The antidiabetic effects of anthocyanins are due to their antioxidant and anti-inflammatory properties and their ability to inhibit the α-amylase, α-glucosidase, DPP-IV enzymes and the activity and overexpression of PTP1B protein [41]. The extract and the isolated compounds effectively improved insulin sensitivity and refinement of insulin resistance [100]. On the other hand, anthocyanin-rich foods were tested in type-2 diabetic patients. All studies reported a decrease in insulin resistance and improved lipid profile [100,101].

Anthocyanins keep β-cell viability and induce insulin release and improve insulin sensitivity via downregulation of the adipocytokine retinol-binding protein 4 (RBP4). In addition, they increase GLUT4 in adipose tissue and skeletal muscle through the activation of the AMPK pathway [44,97,100]. They activate AMPK and modulate genes involved in; insulin-glucose signaling pathways, the expression of PPAR γ, adipocyte-specific genes (lipoprotein lipase) and can up-regulate the gene expression of adiponectin and down-regulate plasminogen activator inhibitor-1 and interleukin-6 genes. [96,99,102].

High anthocyanin intake induced a significant reduction in peripheral insulin resistance detected in females aged 18–76 years [103]. The increase in plasma insulin level and the improvement of glycemic control in type 2 diabetic adults were reported after daily consumption of *Cornus mas* extract for 6 weeks [104,105].

The bioactivities of anthocyanins are different, although they share the same chemical scaffold. The structure–activity relationship influences the bioavailability and bioactivity of anthocyanins. The degree of hydroxylation, methylation, acylation and the sugar moiety attached to make the difference. The in vitro studies showed that the suppression of gut glucose absorption is the major mechanism of anthocyanin anti-diabetic effect through inhibition of α-glucosidase and α-amylase enzymes. The substitution of glucose or galactose enhances the enzyme inhibition activity at the 3-O position or by acylation with ferulic or caffeic acid derivatives [103,105].

The major drawback of anthocyanin use is its poor bioavailability and stability. A new drug design can be used: the encapsulation with protective compounds such as chitosan, cyclodextrin, liposomal micelles or the use of micro or nano-encapsulation. These forms can also offer an extra advantage of controlling the release of the substance. More studies must decide the appropriate consumption and the most effective type as antidiabetic [99,102,103,106].

**Table 6:** Proposed antidiabetic effects of different anthocyanins.

Name of Compound	Chemical Structure	Mechanism	References
Cyanidin	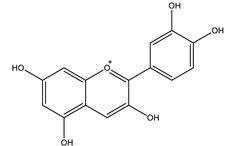	✓ Activates AMPK and GLUT-4 translocation ✓ Decreases gluconeogenesis by reduction of gene expression of PEPCK, G6Pase ✓ Inhibits α-glucosidase and α-amylase ✓ Improves antioxidant status ✓ Prevents pancreatic apoptosis and activating insulin receptor phosphorylation	[41,107]
Delphinidin	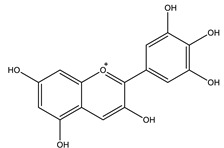	✓ Exhibits anti-inflammatory activity ✓ Inhibits oxidative stress ✓ Inhibits NF-κB and JNK signaling pathways activity ✓ Inhibits protein tyrosine phosphatase 1B PTP1B expression	[41,106]
Pelargonidin	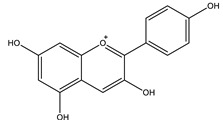	✓ Relieves oxidative stress ✓ Stimulates insulin secretion	[41,108]

**Table 7:** In vitro, in vivo and in silico models of different isolated anthocyanins.

Compound	Mechanism	Dose of the Tested Compound (Duration)	Model	References
Cyanidin	Increased intracellular Ca^2+^ stimulated insulin secretion and the expression of genes involved in this process.	100 µM	In vitro: β-cells INS-1	[109]
Delphinidin	Inhibited glucose absorption by free fatty acid receptor 1 (FFA1).	100 µM	In vitro: Caco-2 and HT-29 cells	[110]
	Inhibited α-amylase Inhibited α-glucosidase.	IC_50_: 601.56 nM IC_50_: 268.41 nM	In vitro: enzymatic assay	[111]
Pelargonidin	Inhibited α-amylase Inhibited α-glucosidase.	IC_50_: 2067.78 nM IC_50_: 175.04 nM	In vitro: enzymatic assay, in silico	[111,112]

#### 8.1.4. Flavonoids

Flavonoids are widespread residents in the plant kingdom with approximately 8000 compounds that support plant development, growth and defense. They share 15 carbon skeletons, and according to the modifications on the generic structure, they are classified into six subclasses [16,41]. Flavonoids have been used for their positive health effects, potential biological functions as antioxidants and treating various human diseases, including diabetes [16].

They are considered promising anti-diabetic agents affecting novel targets such as insulin receptors, protein tyrosine phosphatase, peroxisome proliferator-activated receptor-gamma, and adenosine monophosphate-activated protein kinase (Table 8) [113].

They support the regulation of carbohydrate digestion, glucose metabolism in the liver, insulin signaling, insulin secretion, glucose uptake, adipose deposition, β-cell proliferation and apoptosis (Table 9 and Table 10) [41].

Although flavonoids are known for their poor bioavailability, the invention of advanced delivery techniques (microencapsulation, nano-systems, microemulsions, enzymatic methylation) can overcome the problem [41,114]

The optimal human dietary daily consumption level of flavonoids is not confirmed yet, due to the conflicting bioavailability results and diverse molecular structures. However, flavonoids possible genotoxicity and mutagenicity were reported in mammalian experiments. Fortunately, they are not expected to be induced by dietary flavonoid-rich sources [41,114].

Although few clinical trials on humans were conducted to explore the effect of the flavonoids-rich extract on type-2 diabetic patients, data with promising results were noted. In comparison, individual consumption of flavonoids revealed no fruitful impact on diabetes, while the elevated intake of flavonoids in clinical trials showed a decreased risk of developing diabetes. It is necessary to run more clinical trials using isolated flavonoids or defined standardized flavonoid mixtures to explore the efficacy, pharmacokinetics, and safety profiles [16,114].

Recently, a meta-analysis had specified an association between high intake of total flavonoids and the reduced risk of type-2 diabetes in a dose-dependent manner, specifically for anthocyanidins, flavan-3-ols, flavonols, and isoflavones [115].

Alpha-glucosidase inhibition is a promising target for flavonoid antidiabetic activity. Generally, the 3′ position (ring B) favors bulky and minor electron-withdrawing and hydrogen bond donor groups. At position 4′ (ring B), electron-donating and hydrogen bond acceptor groups improve inhibitory activity. In addition, position 7 (ring A) favors bulky and hydrogen bond acceptor groups [113]. More extensive research is needed to relate the flavonoid structure to its activity and efficacy to identify the involved enzymes, receptors, signaling molecules, and transporter or transcription factors.

**Table 8:** Antidiabetic effects of flavonoids.

	Glucose Transporter	Hepatic Enzymes	Β-Cells Apoptosis	PPAR	AMPK	Tyrosine Kinase Inhibitor	NF-κB
Target	IRS-1	G6pase, FD pase PEPCK, G6PD Hexokinase	Bcl-2 family	Gene expression	AMPK	Tyrosine kinase inhibitors	
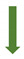 Flavonoid effects 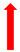	Activation of IRS-1	Insulin signaling Liver glycogen	Apoptosis	--------	--------	Activity of tyrosine kinase	-------
	The synthesis and translocation of GLUT	Hexokinase activity in liver	---------	Expression of PPARγ	AMPK activation	---------	Activity of NF-kB

**Table 9:** Proposed antidiabetic effects of different flavonoids.

Name of Compound	Chemical Structure	Mechanism	References
Apigenin	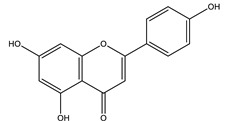	✓ Accelerates insulin secretion and glycogen synthesis ✓ Enhances glucose transporter 4 (GLUT4) translocation ✓ Enhances beta cell preservation ✓ Improves antioxidant parameters	[108,116]
Baicalein	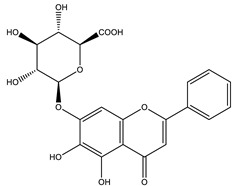	✓ Activates AMPK pathway ✓ Attenuates inflammation and insulin resistance by phosphorylating IRS-1, Akt and dephosphorylate extracellular signal-regulated kinase (ERK), JNK and NF-κB ✓ Inhibits oxidative-nitrosative stress ✓ Increases blood insulin level ✓ Improves β-cell survival ✓ Suppresses fatty acid synthesis and increasing mitochondrial –β oxidation	[33,117]
Chrysin	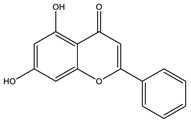	✓ Decreases lipid peroxidation ✓ Decreases oxidative stress and inflammation Increases insulin level ✓ Improves insulin signal transduction	[44,118,119]
Diosmin	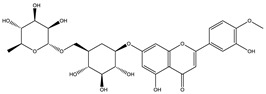	✓ Ameliorates oxidative stress ✓ Enhances the activity of glycolytic enzymes such as hexokinase, glucose-6-phosphate dehydrogenase ✓ Inhibits the gluconeogenic enzymes such as glucose-6-phosphatase and fructose 1,6-bisphosphatase ✓ Increases insulin levels	[108,120]
Daidzein	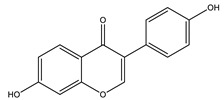	✓ Enhances AMPK phosphorylation in muscles ✓ Improves insulin sensitivity ✓ Inhibits gluconeogenesis through reducing activities of G6Pase, PEPCK, ✓ Improves metabolism of liver lipid ✓ Inhibits α-amylase and glucosidase enzymes	[108,121]
Eriodictyol	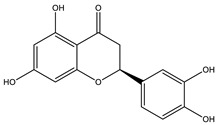	✓ Improves PI3K/Akt pathways ✓ Improves insulin secretion ✓ Increases glucose uptake in tissues ✓ Suppresses of oxidative stress ✓ Up-regulates mRNA expression of PPARγ	[108,118]
Genistein	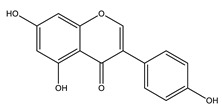	✓ Improves β-cell mass ✓ Inhibits gluconeogenesis through reducing activities of G6Pase, PEPCK, ✓ Improves metabolism of liver lipid ✓ Inhibits the activity of tyrosine kinase ✓ Protects Langerhans islet β-cells by targeting the AKT and ERK1/2 expression and preserving the pancreas via decreasing caspase-3 and increasing anti-apoptotic Bcl-2 protein levels ✓ Reduces blood glucose level through the activity of cAMP, PKA pathway	[41,121]
Fisetin	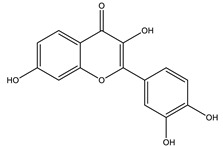	✓ Decreases glycogen breakdown ✓ Decreases protein expression levels of gluconeogenic genes such as phosphoenol pyruvate carboxykinase ✓ Inhibits gluconeogenesis ✓ Increases plasma insulin	[108,119]
Kaempferol	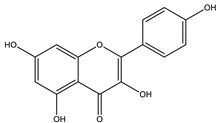	✓ Enhances glucose uptake ✓ Enhances antioxidant production ✓ Inhibit apoptosis ✓ Improves cAMP signaling and enhance insulin synthesis and secretion ✓ Mitigates PPARγ agonist activity ✓ Reduces caspase-3 activity in β- cells	[108,116]
Luteolin	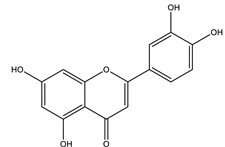	✓ Increases transcriptional activation of PPARγ ✓ Improves insulin secretion	[41,108]
Neohesperidin	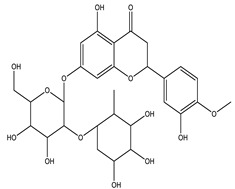	✓ Exhibits antioxidant and anti-inflammatory effects ✓ Activates PPARγ ✓ Enhances expression of GLUT4 adipose tissue ✓ Reduces hepatic glucose output via suppression of PEPCK and G6Pase expression	[33,122]
Naringenin	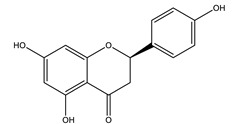	✓ Decreases gluconeogenesis ✓ Inhibits the intestinal α-glucosidase ✓ Improves antioxidant status ✓ Increases glucose uptake ✓ Increases GLUT-4 translocation ✓ Increases activity of AMPK ✓ Increases insulin secretion	[120,123]
Naringin	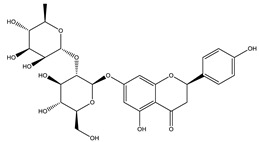	✓ Activates PPAR γ ✓ Accelerates glucose uptake and enhanced AMPK activation ✓ Inhibits DPP-4 enzyme ✓ Protects antioxidant defense system ✓ Reduces hepatic glucose output via suppression of PEPCK and G6Pase expression ✓ Suppress pro-inflammatory cytokine production	[110,120,124]
Morin	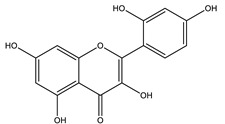	✓ Enhances the phosphorylation of the insulin receptor, Akt, and FOXO1 ✓ Enhances glycogen synthesis ✓ Improves antioxidant activity ✓ Hinders gluconeogenesis ✓ Reduces inflammatory cytokines IL-1β and IL-6 ✓ Recovers of hepatic insulin and leptin sensitivity	[108,116]
Quercetin	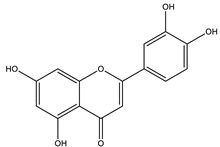	✓ Blunts free radical and oxidative stress ✓ Stimulates glucose transporter type 4 (GLUT4) translocation in skeletal muscle ✓ Inhibits glucose-6-phosphatase (G6Pase) in hepatocytes ✓ Reduces intestinal glucose absorption by inhibiting GLUT2	[35,110,125]
Rutin	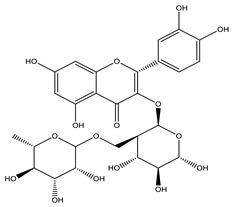	✓ Anti-apoptotic activity ✓ Affects the activities of G6Pase and glycogen phosphorylase ✓ Activates the synthesis and translocation of GLUT-4 in the muscle ✓ Activates the expression of PPAR-γ ✓ Decreases glucose-6-phosophatase, PEPCK, glycogen phosphorylase and fructose -1,6-bisphosphatase ✓ Decreases the level of oxidative stress ✓ Inhibits α-amylase and glucosidase ✓ Increases secretion of insulin ✓ Increases hexokinase activity in liver ✓ Improves the morphology of islets of Langerhans ✓ Increases tissue glucose uptake through activation of mitogen-activated protein kinase (MAPK)	[35,44,110]
Tangeretin	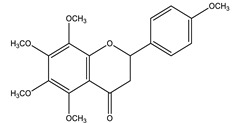	✓ Enhances glucose uptake and GLUT 4 translocation ✓ Improves AMPK signaling	[116,126]

**Table 10:** In vitro, in vivo and in silico models of different isolated flavonoids.

Compound	Mechanism	Dose of the Tested Compound (Duration)	Model	References
Apigenin	Exhibited free radical scavenger and a regulator activity to antioxidant defenses of pancreatic cells.	* ND	In silico	[127]
	Inhibited DPP-4 enzyme.	1.5 mg/kg for every alternate (4 weeks)	High-fat and high fructose-induced diabetic rats, in silico	[124]
Baicalein	Suppressed hepatic gluconeogenesis via activation of the AMPK and AKT signaling pathways.	12.5 mM (24 h)	In vitro: HepG-2 cell line	[128]
	Suppressed expression of PGC-1α (upregulate hepatic gluconeogenic gene expression) and gluconeogenic genes ameliorated hepatic insulin resistance and gluconeogenic activity by inhibiting the p38 MAPK/PGC-1α signal pathway.	50 mg/kg (21 days) 100 µM (12 h)	High-fat diet-induced insulin-resistant mice Primary hepatocytes	[125]
	Promoted glucose uptake through enhancement of GLUT4, PGC-1α, MAPK, AKT and contents.	100, 200, 400 μM (6,12, 24 h)	In vitro: L6 myoblast cell line, C2C12 cell line, animal model	[129,130]
Chrysin	Modified oxidative stress.	20, 40, 80 mg/kg	Streptozocin-induced diabetic rats	[131]
	Activated insulin signal transduction such as IR, IRS-1, Akt	100 mg/kg (4 weeks)	High fat-induced diet diabetic rats	[132]
Eriodictyol	Exerted glucose-dependent insulinotropic effect through cAMP/PKA pathway.	200 μM	In vitro: mice islets and MIN6 cell line	[133]
	Activated glucose utilization, suppressed gluconeogenesis, decreased pro-inflammatory cytokines and increased anti-inflammatory cytokine.	0.05% (*w*/*w*) (16 weeks)	Animal model	[134]
Genistein	Increased insulin level, regenerated β-cells.	10, 20 mg/kg (4 weeks)	Streptozocin-induced diabetic rats	[135]
	Inhibited α-amylase Inhibited α-glucosidase.	IC_50:_ 165.51 nM IC_50:_ 1394.36 nM	In vitro: enzymatic assay	[111]
Fisetin	Inhibited high glucose-induced reactive oxygen radical production through the activation of SIRTs and FOXO3a.	3, 5 and 10 μM (48 h)	In vitro: THP-1 cell line	[136]
Hesperidin	Had antioxidant effect, protective effect for β-cells.	100 mg/kg (15 days)	Streptozocin-induced diabetic rats	[137]
	Decreased oxidative stress and NF-kB levels and increased while SIRT1 level.	100 mg/kg (15 days)	Streptozocin-induced diabetic rats	[138]
	Improved glycogen content by reinstating the activities of glycogen synthase and glycogen phosphorylase.	25, 50, 100 mg/kg (4 weeks)	Streptozocin-induced diabetic rats	[139]
Luteolin	Inhibited high glucose-induced reactive oxygen radical production through the activation of SIRTs and FOXO3a.	3, 5 and 10 μM (48 h)	In vitro: THP-1 cell line	[136]
Morin	Improved insulin signaling through inhibition of microRNA-29a (an essential regulator of insulin signaling and gluconeogenesis pathways).	50 µM (24 h)	In vitro: HepG-2 cell line	[140]
Naringenin	Exhibited antioxidant and anti-inflammatory effects.	* ND	In silico	[127]
	Decreased oxidative stress through promoting nuclear factor E2-related factor 2 (Nrf2), restored insulin expression, promoted glycolysis while inhibiting gluconeogenesis.	50 mg/kg (45 days)	In vitro: MIN6 cell line, streptozocin-induced diabetic rats	[141]
	Improved mRNA expressions of insulin receptor b subunit, GLUT4 and adiponectin.	100 mg/kg (4 weeks)	Streptozocin-induced diabetic rats	[142]
Naringin	Improved mRNA expressions of insulin receptor b subunit, GLUT4 and adiponectin.	100 mg/kg (4 weeks)	Streptozocin-induced diabetic rats	[142]
	Inhibited both intrinsic and extrinsic pathways of β-cell apoptosis, possibly by interfering with DNA damage- and cytokine-induced apoptotic signaling by suppressing pancreatic reactive oxygen species accumulation and leukocyte infiltration.	50, 100 mg/kg (2 weeks)	Streptozocin-induced diabetic rats	[143]
Quercetin	Activated AMPK- MAPK pathway to induce glucose uptake.	10, 100 µM (24 h)	In vitro: L6 myoblast cell line	[144]
	Promoted hepatic glycogen synthesis and reduced blood glucose by increasing Akt phosphorylation, GSK-3 phosphorylation, and GCK protein expression levels.	10, 50 mg/kg (12 weeks)	Streptozocin-induced diabetic rats	[145]
	Exhibited antioxidant effect, protective effect for β-cells.	100 mg/kg (15 days)	Streptozocin-induced diabetic rats	[137]
	Decreased oxidative stress and NF-kB levels and increased while SIRT1 level.	100 mg/kg (15 days)	Streptozocin-induced diabetic rats	[138]
Tangeretin	Exhibited antiapoptotic property due to its inhibitory effect on oxidative stress.	0, 10, 20, and 40 μM (12 h)	In vitro: INS-1 cell line	[146]
	Exhibited a potential insulin action enhancer that functions by inhibiting the MEK-ERK1/2 pathway in hepatocytes.	10,20 mM (48 h) 25, 50 mg/kg (1 month)	Animal model	[147]
	Inhibited α-glucosidase Inhibited α-amylase.	IC_50_ 285.88 nM IC_50_ 682.75 nM	In vitro: enzymatic assay	[111]

ND * not determined.

#### 8.1.5. Stilbenoids

**Figure 13:**
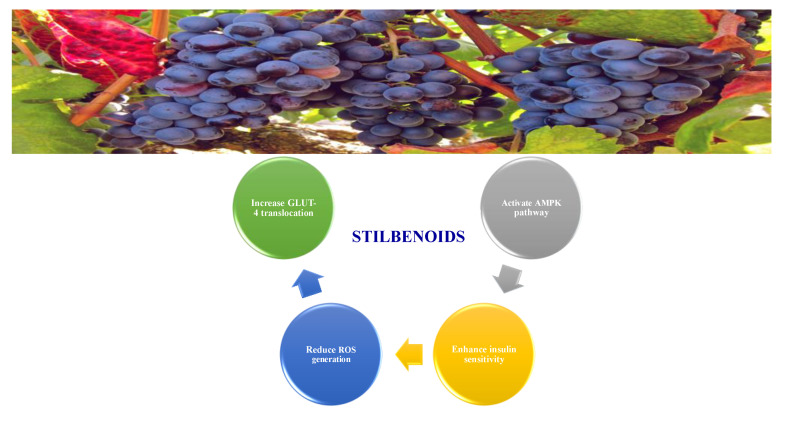
Antidiabetic activity of stilbenoids.

Stilbenoids are antimicrobial compounds produced de novo to protect plants from fungal infection and toxins [148]. They share the same backbone (C6-C3-C6) with different substitutions on the aromatic rings. Dietary stilbenoids are rarely reported as antidiabetic agents except for resveratrol, pterostilbene, and polydatin (Figure 13) [10,148]. Table 11 summarizes the hypoglycemic effect of some dietary stilbenoids. Table 12 shows the studied antidiabetic mechanisms of stilbenoids.

#### 8.1.6. Saponins

**Figure 14:**
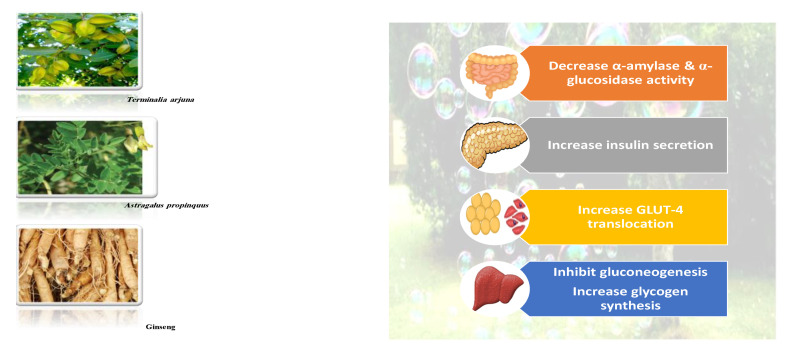
Antidiabetic activities of saponins.

Saponins are bioactive steroidal or triterpenoid glycosides with numerous biological activities and potent antihyperglycemic activity (Figure 14) [15,33].

The antihyperglycemic activity of saponins from various plants was confirmed in vivo and in vitro models [153]. They reduce hyperglycemia through several proposed mechanisms: restoration of insulin response, improvement in insulin signaling, increase plasma insulin levels, induction of insulin release from the pancreas, activation of glycogen synthesis, inhibition of gluconeogenesis, inhibition of α-glucosidase activity, inhibition of mRNA expression of glycogen phosphorylase and glucose-6-phosphatases, increase the expression of Glut4 (Table 13) [153].

Astragaloside IV significantly decreases blood glucose by attenuating insulin resistance in adipocyte cells and inhibiting glycogen phosphorylase and G6Pase [33].

The tetracyclic triterpene structure is pivotal for the antidiabetic activity of saponins. Ginsenosides, from *Ginseng* species, were extensively studied for their hypoglycemic activity. Several animal models and cell lines studies have documented the beneficial effect of ginsenoside through affecting several signaling pathways of glucolipid metabolism. On the other hand, several clinical studies were conducted using ginseng extracts. The results were conflicting. To extend the therapeutic effects, SAR, pharmacokinetic and quality control should be investigated. Table 14 shows studied mechanisms of saponins [149,154,155].

**Table 13:** Proposed antidiabetic effects of different saponins.

Name of Compound	Type of Saponin	Chemical Structure	Mechanism	References
Arjunolic acid	Triterpene saponins	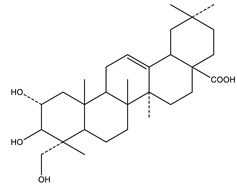	✓ Antioxidant activity ✓ Inhibits α-amylase and glucosidase	[153,156]
Astragaloside IV	Steroidal saponins	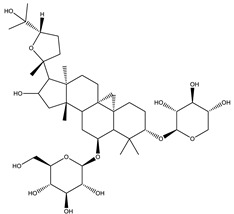	✓ Inhibits hepatic glycogen phosphorylase ✓ Inhibits glucose 6- phosphatase ✓ Improves insulin resistance	[33,153]
Diosgenin	Steroidal saponins	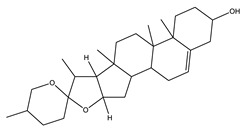	✓ Increases pyruvate Kinase activity (increase hepatic glucose absorption) Decreases insulin resistance ✓ Reduces gluconeogenesis (reduce glucose 6 phosphatase activity) ✓ Inhibits α-glucosidase and absorption (SGLT-1) ✓ Increases GLUT4 translocation level ✓ Increases phosphorylation of Akt ✓ Inhibits glycogen hydrolysis ✓ Promotes insulin secretion	[157,158]
Platyconic acid	Triterpene saponin	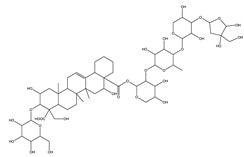	✓ Antioxidant activity ✓ Enhances insulin sensitivity ✓ Increases glycogen synthesis ✓ Promotes PPARγ, PPARα activity ✓ Promotes GLUT4 translocation ✓ Promotes glucokinase, glucose 6-phosphate dehydrogenase	[153,157]
Ginsenoside K	Steroidal saponin	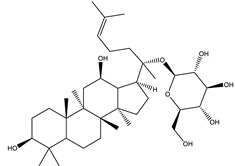	✓ Inhibits gluconeogenesis by down-regulating PEPCK and G6Pase levels ✓ Increases AMPK activation	[33,159]
Ginsenoside Rg3	Steroidal saponin	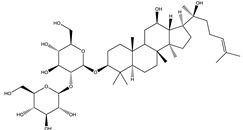	✓ Inhibits inflammatory response ✓ Enhances insulin secretion ✓ Stimulates GLP-1 secretion	[33,160]
Ginsenoside Rg1	Steroidal saponin	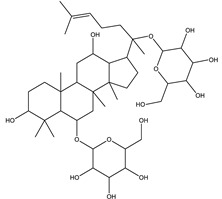	✓ Promotes glucose uptake via activating AMPK pathway in insulin resistance muscle cells ✓ Inhibits gluconeogenesis through decrease transcription of PEPCK and G6Pase ✓ Increase antioxidant enzymes level	[33,161]

#### 8.1.7. Tannins

Tannins are complex polyphenolic compounds of various molecular weights. They are classified as hydrolyzable and condensed tannins. Hydrolyzable tannins are esters of polyol carbohydrate and gallic acid or ellagic acid. Condensed tannins (proanthocyanidins) are oligomers or polymers of polyphenolic bioflavonoids, specifically taking the form of polyhydroxy flavan-3-ol units and flavan-3,4-diols [169,170].

They have several benefits to health, such as free radicals’ scavengers and as activators to antioxidant enzymes. Their benefits encompass the control and management of chronic diseases, including diabetes. They enhance insulin-signaling pathways, such as PI3K (phosphoinositide 3-kinase), p38 MAPK (mitogen-activated protein kinase) activation and GLUT-4 translocation leading to increase glucose uptake [169]. In addition, they reduce the intestinal absorption of glucose and other nutrients, induce β cell regeneration, inhibit α- amylase and α-glucosidase activity and enhance insulin activity on adipose cells (Table 15) [169,170].

#### 8.1.8. Polysaccharides

**Figure 15:**
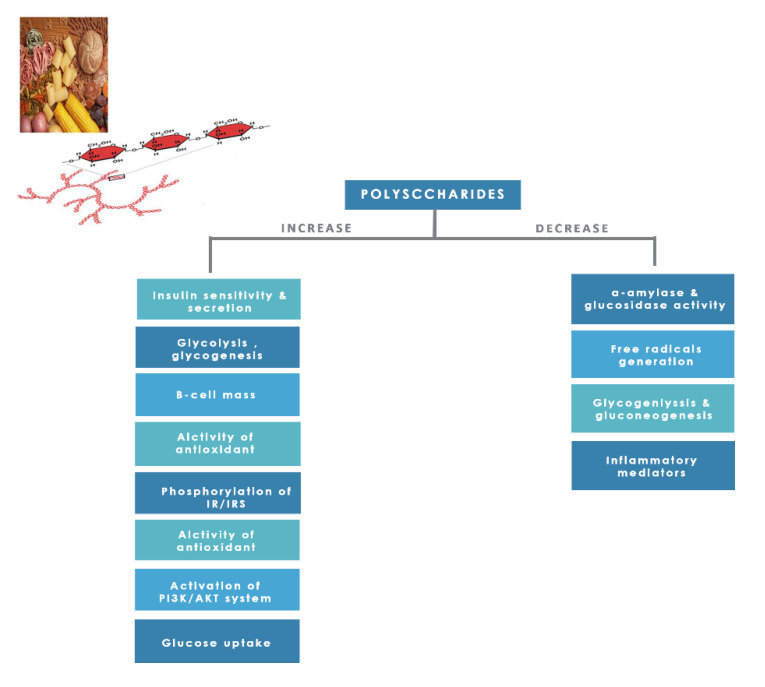
Polysaccharides effects on glucose homeostasis.

Dietary polysaccharides are essential edible compounds for our day-to-day life obtained from plants, grains, fruits, vegetables, and edible mushrooms. Investigators studied them for their numerous pharmacological activities and low toxicity [25]. Polysaccharides showed promising positive effects on human health, such as anticancer, anti-inflammatory action, skin protection, antioxidant, immune modulation, serum cholesterol reduction, and managing diabetes and reducing its complications through several mechanisms (Figure 15) [25,174].

#### 8.1.9. Coumarins

**Figure 16:**
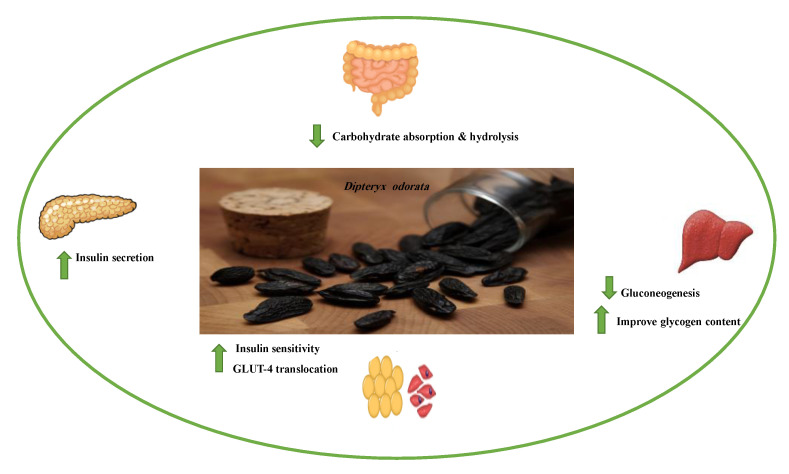
Coumarins effects on glucose homeostasis.

Coumarins are secondary metabolites used widely for their anticoagulation and antithrombotic effects (Figure 16). Abundant in vitro and in vivo studies demonstrated that different coumarin skeletons (simple, furanocoumarin, pyranocoumarin, bicoumarin, and tricoumarin) were good candidates’ against diabetes. Studies have proven that coumarins also exhibit antidiabetic activity through regulating hepatic enzymes, repairing pancreatic β-cells damage, improving insulin signaling, and providing anti-inflammatory and antioxidative properties [157]. Table 16 shows the proposed antidiabetic effects of different coumarins, while Table 17 displays studied antidiabetic mechanisms of coumarins.

Pharmacokinetic profile studies and clinical trials are warranted prior to their use as safe antidiabetics [157]. To enhance their pharmacological potency or pharmacokinetic profile, hybridization between coumarins and other therapeutic pharmacophores opened a new drug method to develop novel hypoglycemic coumarins derivatives. For example, a hybrid molecule of apigenin and coumarin revealed more favorable antidiabetic activity than either compound alone [175].

**Table 16:** Proposed antidiabetic effects of different coumarins.

Name of Compound	Chemical Structure	Mechanism	References
Esculetin	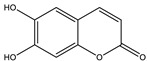	✓ Enhances the activity of fructose-1, 6-biphosphatase and glucose-6-phosphatase ✓ Improves glucokinase activity	[157,176]
Fraxetin	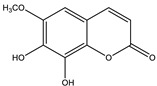	✓ Exhibits antioxidant activity ✓ Improves hepatic glycogen content ✓ Inhibits gluconeogenesis ✓ Improves glucokinase activity ✓ Increases the activity of glucose-6-phosphate dehydrogenase	[157,176]
Umbelliferone	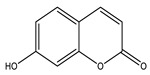	✓ Enhances insulin sensitivity and promote GLUT4 translocation through activation of PPARγ ✓ Improves insulin secretion ✓ Inhibits α-glucosidase ✓ Modulates hepatic lipid metabolism ✓ Reduces adiponectin	[177]

**Table 17:** In vitro, in vivo and in silico models of different isolated coumarins.

Compound	Mechanism	Dose of the Tested Compound (Duration)	Model	References
Esculetin	Improved insulin resistance by increasing hepatic GLUT2 and glucokinase mRNA levels and decreased glucose-6-phosphatase mRNA level.	0.02%, *w*/*w* (12 weeks)	C57BL/6J mice high-fat diet diabetic mice, liver histological model	[178]
	Boosted Akt activation and promoted glucose uptake.	40 mg/kg (14 days)	Dexamethasone-induced insulin resistance mice, C2C12 cell line	[179]
Fraxetin	Boosted Akt activation and promoted glucose uptake.	40 mg/kg (14 days)	Dexamethasone-induced insulin resistance mice, C2C12 cell line	[179]
Osthole	Increased GLUT4 mRNA expression in skeletal muscle.	5–10 mg/kg (6 weeks)	High-fat and high-sucrose induced fatty liver with IR rats	[180]
	Boosted Akt activation and promoted glucose uptake.	20 mg/kg (14 days)	Dexamethasone-induced insulin resistance mice, C2C12 cell line	[179]
Scopoletin	Stimulated GLUT-4 translocation through activation PI3K and AMPK pathway.	1, 2.5, 5, 10, 15, 20 (24 h)	3T3-L1 adipocyte cell lines	[181]
	Inhibited carbohydrate digestive enzymes. Inhibited α-amylase Inhibited α- glucosidase	IC 50: 37.36 µM IC 50: 85.12 µM	In vitro study	[182]
Umbelliferone	Stimulated muscle glucose uptake and stalled gluconeogenesis and oxidative stress.	30–240 µg/mL (2 h)	Ex vivo: isolated psoas muscles	[177]
	Shunted gluconeogenic enzymes, regeneration of the β-cells.	100 mg/kg (4 weeks)	Alloxan-induced diabetic rat	[183]

#### 8.1.10. Terpenes

**Figure 17:**
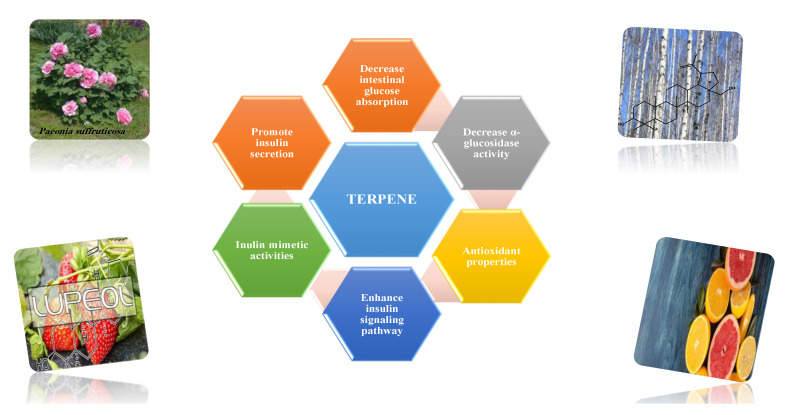
Antidiabetic activity of terpenes.

Terpenoids are secondary metabolites constructed from repeating units of isoprene with wide structural diversity [184]. They are categorized into hemiterpenes, monoterpenes, sesquiterpenes, diterpenes, sesterpenes, triterpenes, tetraterpenes, and polyisoprenes (Figure 17). Terpenes and their derivative prevent various diseases and have many valuable properties [185].

Generally, terpenes showed an antidiabetic effect in both in vitro and in vivo studies. They have excellent antioxidant activity and potent α-glucosidase inhibitory activity except for the monoterpenes lacking the phenolic nature in their structures. In addition, they enhanced insulin level and glucose uptake in tissues and can inhibit several signaling pathways in carbohydrate metabolism [184,185]. Several monoterpenes can facilitate glucose uptake via upregulation of the glucose transporter (GLUT4) translocation, enhance insulin signaling pathway, promote insulin secretion, protect pancreatic cells and ameliorate proinflammatory cytokines [184]. In addition, they increase the level of glycogen levels, modulate glucose-6-phosphatase and fructose-1,6-bisphosphatase, reduced glucokinase and glucose-6-phosphate dehydrogenase activities [33,184]. Table 18 shows proposed antidiabetic effects of different terpenes, and Table 19 shows studied antidiabetic mechanisms of terpenes.

Sesquiterpenes are efficacious than other terpenes due to their tightly packed structure. On the contrary, triterpenes with more hydrophilic groups are more preferable to multi-target effects as antidiabetics [185].

Some terpenes were evaluated clinically, but further randomized studies are needed to confirm the declared antidiabetic activity. Additionally, evaluation for pharmacokinetic parameters and safety profiles are needed [185].

**Table 18:** Proposed antidiabetic effect of different terpenes.

Name of Compound	Class of Compound	Chemical Structure	Mechanism	References
Bassic acid	Triterpene	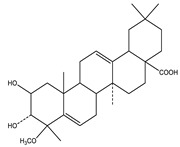	✓ Enhances secretion of insulin	[185]
Limonene	Monoterpene	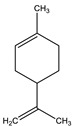	✓ Increases the antioxidant activity ✓ Increases activity of the glycolytic enzyme ✓ Stimulates insulin secretion	[185,186]
Stevioside	Diterpene	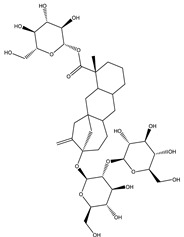	✓ Insulinotropic ✓ Glucagonostatic ✓ Preserves β-cells	[185,187]
Rebaudioside	Diterpene	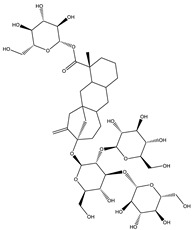	✓ Insulinotropic activity ✓ Increases glycolysis ✓ Inhibits gluconeogenesis	[185,188]
Lupeol	Triterpene	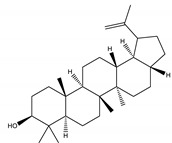	✓ Decreases oxidative stress ✓ Inhibits the activity of PTP1B ✓ Inhibits α-amylase and glucosidase	[185,189]
Palbinone	Triterpene	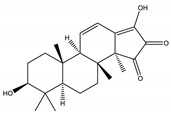	✓ Induces glucose uptake via AMPK pathway	[185,190]
Betulin	Triterpene	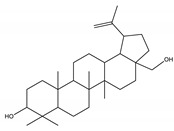	✓ Inhibits α-glucosidase ✓ Induces glucose uptake via activation AMPK pathway ✓ Stimulates mRNA expression of glucose transporter-4 ✓ Increases insulin secretion ✓ Increases muscle glycogen ✓ Potentiates β-cell mass and function	[50,190,191]

**Table 19:** In vitro, in vivo and in silico models of different isolated terpenes.

Compound	Mechanism	Dose of the Tested Compound (Duration)	Model	References
Asiatic acid	Exhibited antioxidant activity Increased insulin secretion in rats with sufficient insulin-secreting function Enhanced glucose uptake into skeletal muscle via PI3K-Akt signaling pathway.	20 mg/kg (45 days)	Streptozocin-induced diabetic rats	[192]
Carvacrol	Increased activity of hexokinase, citrate synthase and 6-phosphofructokinase.	10, 20 mg/kg (4, 6 weeks)	Streptozocin-induced diabetic mice	[193]
Limonene	Exhibited antioxidant activity.	100 mg/kg (8 weeks)	Alloxan-induced diabetic rats	[194]
Stevioside	Increased level of GLUT-4.	1–100 µM (24 h)	Rat L6 myoblast and mouse 3T3-L1 fibroblast cell lines	[195]
	Increased leptin level Exhibited antioxidant activity, Restored normal pancreatic cell function Increased pyruvate kinase expression and insulin receptor substrate-1.	300 mg/kg (4 weeks)	Streptozocin-induced diabetic rats	[196]
Lupeol	Shunted gluconeogenic enzymes regenerated o β-cells.	200 mg/kg (4 weeks)	Alloxan-induced diabetic rat	[183]
	Inhibited carbohydrate digestive enzyme.	10 mg/kg	Streptozocin-induced diabetic mice	[191]
	Regulated insulin receptor and GLUT-4 protein expression in muscular tissue.	25 mg/kg (30 days)	High-fat diet and sucrose-induced diabetic mice	[197]
Lupeol	Controlled insulin signaling molecules such as IR and GLUT2 protein expression in hepatocytes.	25 mg/kg (One month)	High-fat diet diabetic rats	[198]
Betulinic acid	Exhibited pancreatic islet regenerative effects.	10,20, 40 mg/kg (2 weeks)	Streptozocin-nicotinamide-induced diabetic mice	[199]
	Improved the level of leptin and adiponectin.	10, 20, 40 mg/kg	Streptozocin-nicotinamide-induced diabetic mice	[200]
	Enhanced AMPK phosphorylation, stimulated mRNA expression of glucose transporter 4.	200 mg/kg	Alloxan- induced diabetic rats	[49]
	Inhibited α-glucosidase.	IC_50_: (1.06 ± 0.02) ×10^−5^ mol/L	In vitro: enzymatic assay	[201]
	Increased basal glucose uptake.	5, 10 µM (4 days)	HepG2 and 3T3-L1 cell line	[202]
Oleanolic acid	Inhibited α-glucosidase.	IC_50_: 10.11 ± 0.30 µM	In vitro: enzymatic assay	[203]
Ursolic acid	Increased GLUT-4 translocation, increased muscle glycogen content, increased insulin secretion	0.1, 1 and 10 mg/kg	Hyperglycemic rats	[204]
Thymol	Exhibited Antioxidant activity.	40 mg/kg (28 days)	Streptozocin-induced diabetic rats	[205]

## 9. Summary

The management of diabetes is a complicated issue. There is a struggle to lower blood glucose, decrease insulin levels and prohibit insulin resistance. Diabetes is a multifactorial disease, requiring multifactorial strategies to be treated. Although oral antidiabetics and insulin are the keystones for treatment, many obstacles necessitate the investigation for other opportunistic medications. A gastrointestinal burden such as stomach pain and diarrhea are common side-effects for α-glucosidase inhibitors. Thiazolidinediones may cause weight gain, heart failure, and anemia. Other effects, including headache, hypoglycemia, dizziness, nausea and vomiting, fatigue, dyspepsia and genitourinary tract infections, have also been accounted for by conventional anti-diabetic medications. Today, to reverse disease progression by finding a newer drug with novel targets is the main goal to treat diabetes efficiently [42,206,207,208].

Nature is always the savior; animals, microorganisms, marine life and plants all are sources of efficacious and safer drugs. Prospectively, applying natural products in various forms in the healthcare system is extraordinarily bright and urgent. Plant-based medicine is popular, effective, safe, available, and less expensive, serves as a template for drugs and helps detecting new potential targets to treat the disease. In addition, natural products act by affecting multiple targets simultaneously [100,209].

Through various mechanisms of action, plant extract such as *Allium cepa*, *Aloe vera*, *Alium sativum*, *Catharanthus roseus*, *Eucalyptus globules*, *Trigonella foenum-graecum*, *Urtica dioica*, and isolated molecules such as berberine, ginsenosides, curcumin, stevioside, simple phenolic compounds, anthocyanins, resveratrol, genistein and hesperidin are used for remediation of diabetes [210,211]. Asteraceae, Euphorbiaceae, Fabaceae, Lamiaceae, Liliaceae, and Poaceae are rich in anti-hyperglycemic plant genera [210].

Plant secondary metabolites were reported to inhibit carbohydrate metabolizing enzymes, possess kinase activating capacity, thereby affecting all the metabolic pathways of carbohydrate, lipid and protein, and can intervene in the insulin-signaling pathway, inflammatory response, and oxidative stress and restore molecular aberrations leading to insulin resistance and glucose intolerance [22,27].

The phenolic extracts and pure polyphenols may have different effects on the activity of α-amylase and α-glucosidase. For instance, anthocyanins and flavonols have in vitro α-glucosidase inhibition activity, while flavan-3-ols are responsible for α-amylase inhibitory activity. Simple phenolic acid possesses both in vitro α-glucosidase and α-amylase inhibition. The in vitro antidiabetic activity of saponins is affected by the configuration of the C-23 on the side chain and the type of the molecule attached at the C-3. Moreover, polyphenols can inhibit both enzymes in vivo and influence glucose transporter (SGLT1 and GLUT2), augmenting the postprandial hyperglycemia reduction [212,213].

On the contrary, polyphenol can promote glucose uptake by functional tissues through GLUT4 transporter. Berberine, gallic acid, daidzein, resveratrol and vanillic acid can accelerate glucose transportation by AMPK and PI3K-Akt dependent pathways [213,214].

Enhancement of insulin failure to trigger signal transduction in tissues and the restoring of insulin sensitivity by polyphenols were reported to be mediated by activating the AMPK pathway in many in vivo and in vitro studies. Recently, AMPK was proposed as a major drug target to treat diabetes [20,103]. Its activation prompted upregulation of GLUT-4 and enhancement of glucose uptake, reduction in hepatic glucose production and increase fatty acid oxidation. It had been reported that triterpenes stimulate glucose uptake and glycogen synthesis through AMPK activation [49,97].

Recently, PTP1B was considered a potential target for diabetes. As selective inhibitors of PTP1B, ertiprotafib, trodusquemine and claramine reached clinical trials. Trodusquemine is still in phase 2 clinical trials, while ertiprotafib clinical trials were terminated due to the lack of significant clinical efficacy and undesirable side effects. Alkaloids, flavonoids, phenolic acids, steroids, tannins and terpenes can all inhibit the negative regulation of PTP1B [35,36].

Apigenin, kaempferol, luteolin and quercetin are already known PTP1B inhibitors [215,216]. The screening results strongly revealed the important pattern of substitutions on the main flavonoid skeleton. A hydroxyl group on position 3 (ring C), methoxy groups at 3՝ and 4՝ positions (ring B) and the presence of a less polar group at 7 and 8 positions (ring A), arrangement number and characterization of sugar moiety can affect the activity. Flavonols were reported to possess a potent PTP1B inhibitory effect depending on multiple non-polar substituents. In addition, flavanones were described as significant PTP1B inhibitors. They were associated with the presence of a hydroxyl group at position 5 of ring A and the presence of the isoprenyl group [216,217].

Targeting pancreatic β-cells is a new source for successful antidiabetic agents. For example, berberine and quercetin showed a regeneration power of pancreatic β-cells. Oleanolic acid glycoside, geraniol, kaempferol had an anti-apoptosis effect. Resveratrol increased insulin secretion, and vidoline protects β-cells from cytokines induced apoptosis.

Antioxidant power is a preliminary factor for the prediction of antidiabetic activity [218]. NADPH oxidase inhibitors—such as anthocyanins, xanthine oxidase inhibitors—such as catechin and gallic acid, and SOD mimetics can neutralize free radical generation [219,220,221,222].

In summary, secondary metabolites are excellent research candidates to reduce blood glucose levels as they are digestive enzyme inhibitors, insulin secretagogue, insulin sensitizers, insulin signal transductors and insulin modulators.

## 10. Conclusions

This review highlighted the scientific evidence of the plant-derived secondary metabolites as potential multi-target antidiabetic agents. They can modulate enzymes, proteins, signaling pathways, cell proliferation, inflammatory mediators, gene transcription and expression. Numerous compounds are promising antidiabetic agents; however, large scale anti-diabetic evaluation is needed. Therefore, it is important to advocate further research to validate and optimize targets, animal models, toxicity, efficacy, pharmacokinetic studies and clinical trials for multi-target hypoglycemic therapy. Computer-aided research may ease the road for the development of more potent and selective plant-based antidiabetic molecules.

## Figures and Tables

**Table 1:** Effects of insulin on several organs and proteins.

Target		Glucose Transporter	Hepatic Enzymes	Β-Cells Apoptosis	PPAR	AMPK	Tyrosine Kinase Inhibitor	NF-κB
Normal effects		Enhance glucose uptake by tissues	Enhance glucose metabolism	Control program cell death	Control of lipid metabolism	Energy homeostasis	Control growth factor signaling	Control of β-cells survival
Changes by diabetes	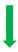	GLUT translocation Glucose uptake	Insulin signaling Liver glycogen	Apoptotic regulatory genes Caspases	Lipid metabolism	AMPK activation Glucose homeostasis	Insulin sensitivity Insulin secretion	
	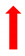	Insulin resistance	Insulin resistance Glucose production	Oxidative stress Insulin resistance Mitochondria dysfunction	Hyperglycemia Hyperlipidemia hyperinsulinemia	Insulin resistance	Islet cell function	Proinflammatory cytokines Oxidative stress NF-κB expression

**Table 11:** Proposed antidiabetic effects of different stilbenoids.

Name of Compound	Chemical Structure	Mechanism	References
Resveratrol	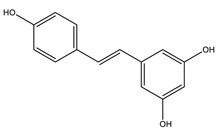	✓ Decreases the expression of pro-inflammatory cytokines ✓ Enhances the activity of antioxidant enzymes ✓ Increases fatty acid oxidation ✓ Increases the activity of hexokinase and pyruvate kinase ✓ Increases hepatic glycogen content by activating glycogen synthase and inhibiting glycogen phosphorylase ✓ Increases GLUT-4 translocation in muscles via PI3K-Akt ✓ Reduces the activity of phosphoenolpyruvate carboxykinase, lactate dehydrogenase, and glucose-6-phosphatase	[10,102,149]
Pterostilbene	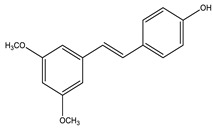	✓ Ameliorates insulin sensitivity ✓ Decreases gluconeogenic enzymes ✓ Decreases proinflammatory cytokines ✓ Enhances antioxidant signaling pathways ✓ Increases the expression of the glycolytic enzyme hexokinase ✓ Increases GLUT-4 translocation ✓ Inhibits digestive enzymes ✓ Protects pancreatic 𝛽-cell ✓ Reduces oxidative stress ✓ Promotes glucose uptake	[10,150]

**Table 12:** In vitro, in vivo and in silico models of different isolated stilbenoids.

Compound	Mechanism	Dose of the Tested Compound (Duration)	Model	References
Resveratrol	Suppressed oxidative stress and increased potential to internalize glucose by extrahepatic tissues.	20 mg/kg (8 weeks)	Streptozocin-induced diabetic rats	[151]
Pterostilbene	Ameliorated morphological impairment of the pancreas, increased the protein expression of PPARγ, PI3K, p-Akt, GLUT4 and IRS-1 in adipose tissues.	20, 40, 80 mg/kg (8 weeks)	Streptozocin-induced diabetic rats	[150]
	Activated Nrf2, thereby reducing oxidative damage, reverted hexokinase, glucose-6-phosphatase, glucose-6-phosphate dehydrogenase, and fructose-1,6-bisphosphatase, to near-normal levels, improved insulin secretion.	5, 10 mg/kg (5 weeks)	In vitro: MIN-6 cell line, streptozocin-induced diabetic rats	[152]

**Table 14:** In vitro, in vivo and in silico models of different isolated saponins.

Compound	Mechanism	Dose of the Tested Compound (duration)	Model	References
Ginsenoside K	Inhibited the expression of PEPCK and G6Pase enzymes, increased the activation of AMPK.	1, 2, 4, 8 μM, (24 h) 30 mg/kg, (4 weeks)	In vitro: HepG2 cell line, streptozocin-induced diabetic mice	[162]
	Inhibited inflammation and improved insulin signaling in adipose tissue by suppressing ER stress-associated NLRP3 inflammation activation.	10 µM (24 h)	In vitro: T3-L1 cell line, mice model	[163]
Ginsenoside Rb1	Increased GLUT-4 translocation through up-regulated adipoR1 and adipoR2 gene.	0.001–100 mM (1–12 h)	In vitro: C2C12 myotubes cell line	[164]
	Exhibited insulin-sensitizing effect.	20 mg/kg (14 days)	Diabetic mice	[165]
	Reduced hepatic glucose production, increased glucose uptake in skeletal muscle.	10 mg/kg every other day (One week)	High-fat-induced diabetic mice	[166]
	Inhibited inflammation and improved insulin signaling in adipose tissue by suppressing ER stress-associated NLRP3 inflammation activation.	10 µM (24 h)	In vitro: T3-L1 cell line, mice model	[163]
Ginsenoside Rg1	Reduced inflammation by inhibiting JNK activity, reduced caspase-3 and BAX (proapoptotic) proteins, increased BCL-2 (antiapoptotic) protein.	25, 50 mg/kg (4 weeks)	Streptozocin-induced diabetic rats	[167]
Ginsenoside RK3	Inhibited hepatic gluconeogenesis (inhibited PEPCK and G6pase protein expressions).	10, 30, 60 mg/kg (4 weeks)	In vitro: HepG2 cell line, high-fat diet and streptozocin-induced mice	[168]

**Table 15:** Antidiabetic effects of different tannins.

Name of Compound	Mechanism	References
Tannic acid	✓ Induces phosphorylation of insulin receptor and Akt ✓ Inhibits α-amylase and α-glucosidase ✓ Promotes GLUT-4 translocation	[170,171]
Condensed tannins	✓ Attenuates oxidative stress ✓ Decreases β-cell apoptosis ✓ Inhibits α-amylase and α- glucosidase ✓ Increases normal insulin content	[172]
Hydrolyzable tannins	✓ Inhibits α-amylase and α-glucosidase	[173]
